# Bioactive and Ion-releasing materials in minimum intervention dentistry: a clinical pathway from prevention to restorative treatment

**DOI:** 10.3389/fdmed.2026.1739208

**Published:** 2026-04-08

**Authors:** Hervé Tassery, Salvatore Sauro, Amel Slimani

**Affiliations:** 1 Ecole de Médecine Dentaire de Marseille, Université d'Aix-Marseille, Marseille, France; 2 Institut Hospitalo-Universitaire Méditerranée-Infection, Aix-Marseille Univ, IRD, MEPHI, AP-HM, Marseille, France; 3 Dental Biomaterials and Minimally Invasive Dentistry, Department of Dentistry, Cardenal Herrera-CEU University, CEU Universities, Valencia, Spain; 4 Department of Therapeutic Dentistry, I.M. Sechenov First Moscow State Medical University, Moscow, Russia; 5 LBN, Univ Montpellier, Faculty of Dentistry, Montpellier, France; 6 CSD Madeleine-Françoise Calais, CHU Montpellier, Montpellier, France

**Keywords:** bioactive dental concept, caries activity, caries risk susceptibility assessment, ions released biomaterials, MI dentistry

## Abstract

**Introduction:**

This paper examined the clinical applications of “bioactive” dental materials within a framework of minimum intervention dentistry, ranging from non-invasive to invasive procedures for managing carious lesions. Modern approaches to caries management emphasise personalised care, caries risk assessment, and lesion activity evaluation, combined with the strategic use of ion-releasing biomaterials (IRB). These materials, which can release ions such as calcium, phosphate, fluoride and other ions, may support dental remineralization, stabilize collagen, buffer pH, and deter bacterial growth, thus promoting long-term oral health.

**Discussion:**

The Bioactive Dental Concept finds a great ally with the new generation ABRAM (Advanced Bioactive Restorative Adhesive Material) and available materials; it is structured to guide clinicians to select bioactive options when clinically appropriate. Interventions are categorised as non-invasive (home care and professional applications), micro-invasive (sealants, resin infiltration, peptides), and invasive. Diagnostic tools like fluorescence and infrared imaging improve early detection, enabling minimally invasive, targeted treatments. Early lesions are sealed and remineralised; moderate ones need selective excavation and cavity conditioning. Extensive lesions require durable bioactive restorations, with new materials like “alkasite” and dual-cure bioactive composites. Integrating evidence-based dentistry (EBD) principles remains essential, though reliance on strict hierarchies of evidence may limit the adoption of innovative approaches. Practical clinical experience and biological acceptability must also inform decision-making.

**Conclusion:**

The article emphasises the need for a balanced approach that combines the rigor of EBD with clinical pragmatism. Even though no restorative material is yet biologically ideal, bioactive and ion-releasing materials significantly improve the management of carious lesions, supporting the aim of preserving natural tooth structures and promoting oral eubiosis.

## Key points

The article aimed to provide an overview of bioactive products and clinical interventions beneficial for managing carious lesions, with restorative procedures systematically categorized as non-invasive, micro-invasive, and invasive techniques.

## 1 Introduction

A modern, person-focused, team-based approach to carious lesion management has emerged from a better understanding of how these lesions form, resulting in new, targeted treatments. These therapies emphasise using bioactive materials appropriately and do not always require operative intervention. Even Care should follow the minimum intervention concepts like CAMBRA, MIOC or CariesCare 4D and incorporate the Bioactive Dental Concept (1–5), which could promote the use of new ions-releasing biomaterials. Such materials are defined as products that in contact with biological fluids can trigger a biological response, forming bonds with tissues via apatite formation. Others consider bioactive materials those able to release specific ions capable of binding collagen, promoting mineral formation, protecting collagen, maintaining a favourable pH, and deterring bacteria. This paper examined the clinical applications of “bioactive” dental materials within a framework of minimum intervention dentistry, ranging from non-invasive to invasive procedures for managing carious lesions. The hypothesis is that modern approaches to caries management should emphasise personalised care, caries risk assessment, and lesion activity evaluation, combined with the strategic use of ion-releasing biomaterials (IRB) (1, 2).

## 2 Steps of the minimum intervention concept and bioactive dental concept

### 2.1 Definition of the word “bioactive” material

Larry Hench has described a bioactive material as one that elicits a specific cellular and biological response at the interface of the material, which results in the formation of a bond between the tissues and the material or one that forms a surface layer of an apatite-like material in the presence of saliva or a saliva-like substitute (3). The vast majority of biomaterials in dentistry do not meet this “bioactive” definition; others use the term Dental Bioactive Material or Ions Released Biomaterials, which is ideally able to bind to collagen, acting as a template of calcium and phosphorus and stimulating the nucleation of apatite crystallization, protecting collagen from degradation, providing an adequate pH to favour new mineral formation, having a pH buffering effect to reduce the secondary caries risk and repelling or constraining bacteria (2, 4).

### 2.2 Minimum intervention concept

Several key principles outlined in this chapter underpin the concept of minimum intervention dentistry. First, individual caries risk and susceptibility should be assessed longitudinally using validated frameworks such as CAMBRA (Caries Management by Risk Assessment), MIOC (Minimum Intervention Oral Care), and CariesCare4D (5–7). Patients should receive individualized counselling on oral hygiene practices and dietary habits, with particular attention to the amount, frequency, and duration of consumption of sugary and acidic foods throughout the day. Based on this comprehensive assessment, a personalized care plan should be developed, incorporating non-invasive, micro-invasive, and, when necessary, minimally invasive operative interventions in line with the MIOC framework. Additional practical guidance can be found in the UK evidence-based prevention toolkit, Delivering Better Oral Health (8). The use of the ICCMS™ (9, 10) *(International Caries Classification and Management System*) classification is recommended. Prevention and disease control strategies should be implemented within a holistic, patient-centred preventive framework; including non-invasive, micro-invasive, and invasive interventions, as well as patient-directed behavioural modifications. Minimally invasive operative dentistry further involves the development and use of sonic or ultrasonic instrumentation (11) with inserts specifically adapted to the size and morphology of the cavity (12), thereby preserving sound tooth structure. Finally, active surveillance protocols are essential to reduce the risk of secondary caries. This approach supports the maintenance of a balanced oral microbiota and a non-cariogenic biofilm, consistent with the Eubiosis paradigm (13, 14).

### 2.3 Specificity of the bioactive dental concept

Clinical decision-making is guided by three principal parameters: the presence or absence of cavitation, the activity status of the lesion, and the accessibility of the carious lesion, particularly in terms of its cleanability (15). In addition, the use of IRB, adapted according to lesion location and patient-specific factors, may reasonably inform management when these three clinical criteria are considered. Integrating these elements, together with complementary clinical and biological considerations, further refines and completes the bioactive dental concept.

#### 2.3.1 Three clinical situations

First, patient with a High Individual Caries Risk, priority should be given to rebalancing the caries risk profile through targeted preventive and therapeutic strategies (6, 16). Definitive operative decisions should follow only after this risk modulation has been addressed.

Second, in the presence of any active dentinal carious lesion, management should aim to promote lesion arrest or reversal. This includes leveraging the buffering capacity of restorative materials and facilitating the formation of an interdiffusion zone with remineralization potential at the tooth–material interface (17).

Third, in cavities with little or no bondable enamel at the gingival margins, particularly when associated with active carious dentin, material selection and operative strategy must account for the compromised substrate and the biological activity of the lesion to optimize marginal integrity and long-term stability (18).

#### 2.3.2 Additional key points

Before diagnosing suspicious surface lesions, thorough biofilm removal is essential. Airflow technology may be used according to the intended level of intervention: erythritol powder for non-invasive cleaning; sodium bicarbonate or calcium carbonate powders for micro-invasive procedures; and bioactive glass powders when a more invasive tooth-cleaning approach is indicated. The use of rotary brushes with prophylactic paste should be avoided. Their bristle diameter (approximately 0.2 mm) is too large to effectively clean narrow pits and the deepest areas of fissures and residual paste may interfere with certain diagnostic devices. In cases of proximal lesions, placement of a tooth separator, such as the Ivory tooth separator, can facilitate visual access and improve operative management. The application of disclosing or fluorescent agents may also provide additional information for plaque-detection systems (6). The use of dedicated diagnostic technologies (Figures 2, 3) can further support lesion assessment and guide selective caries removal. This approach prioritizes preservation of leathery dentin while respecting the principle of the peripheral seal zone, thereby ensuring an effective marginal seal and protecting the integrity of the dentin–pulp complex (19, 20). Subsequently, the application of IRB, based on caries risk susceptibility, lesion activity, availability of bondable enamel at the gingival margins, and lesion accessibility/cleanability (18, 21), provides a rational framework for clinical decision-making (see Supplementary Tables S9, S10). Finally, continued appraisal of the current level of evidence using the Oxford Centre for Evidence-Based Medicine (OCEBM) framework remains necessary to refine and further develop these techniques for the benefit of patients (see Supplementary Table S11).

**Figure 1:**
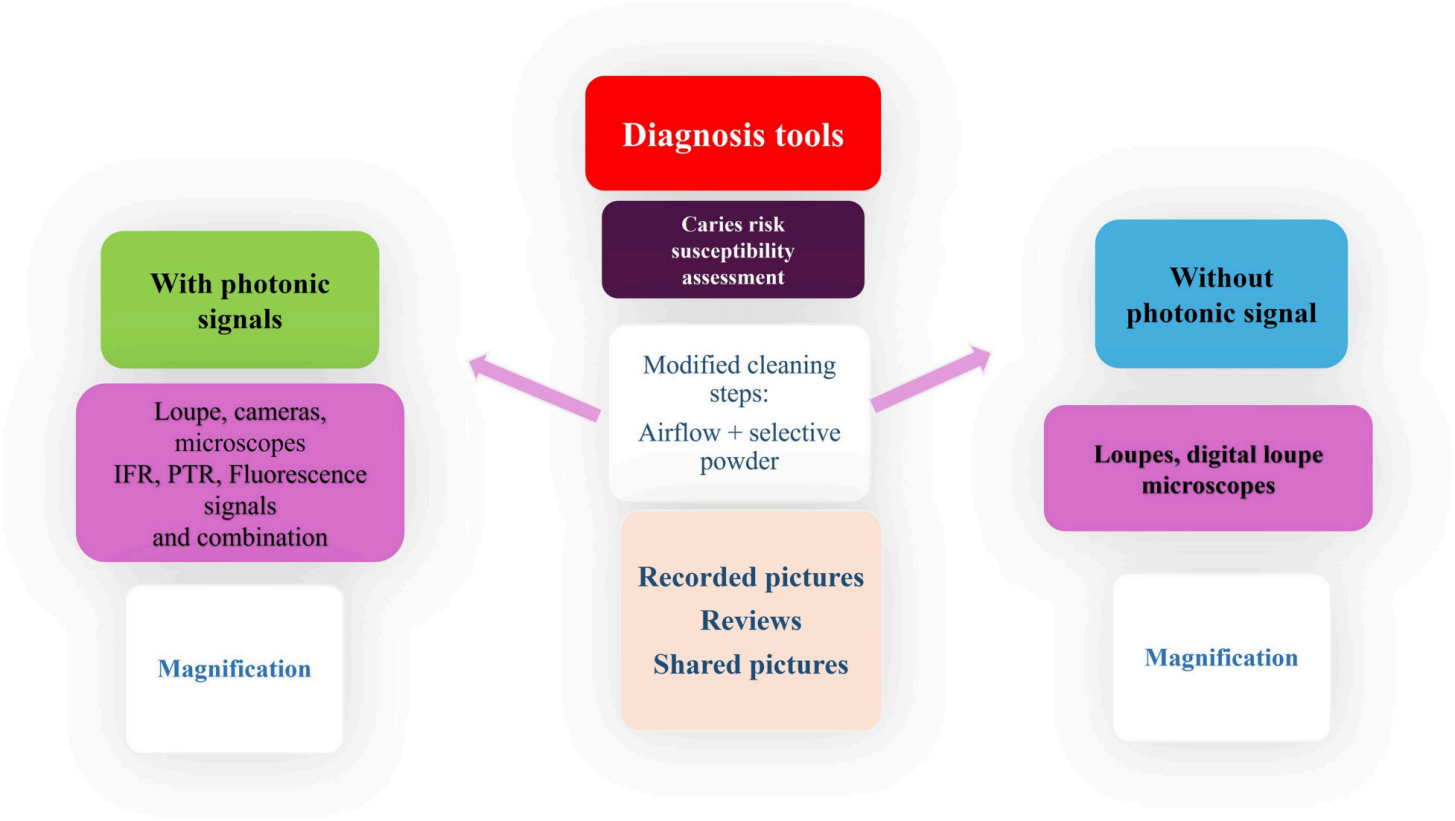
Expert-driven flow chart for modern caries detection technology. It includes photonic signals (fluorescence, Infrared (IFR), and photothermal radiometry (PTR).

**Figure 2:**
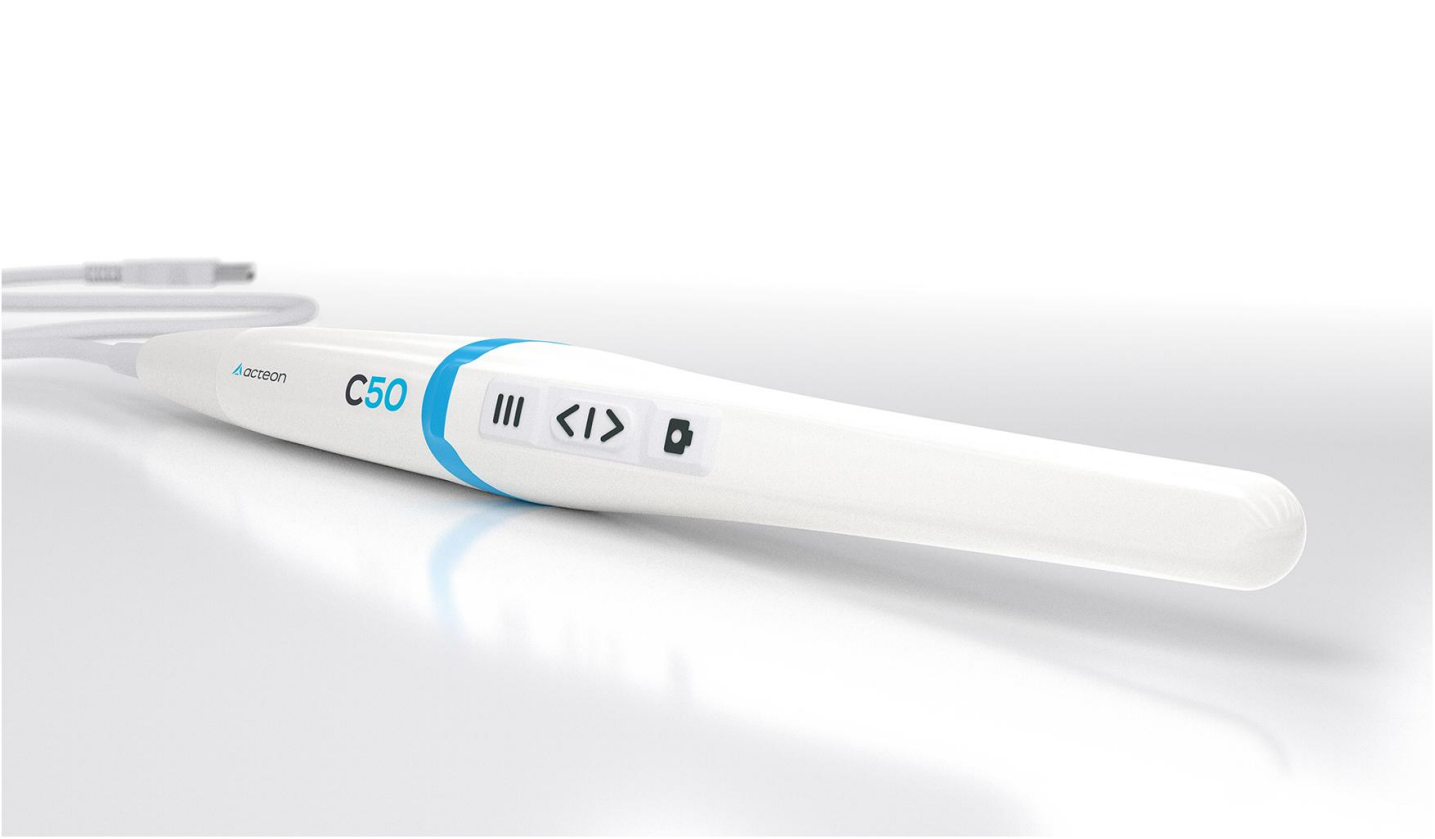
Newly released intraoral C50® camera (acteon, France) with 2 daylight modes (classic and boosted) and 2 fluorescence modes (caries and periodontal modes) for dental diagnosis.

**Figure 3:**
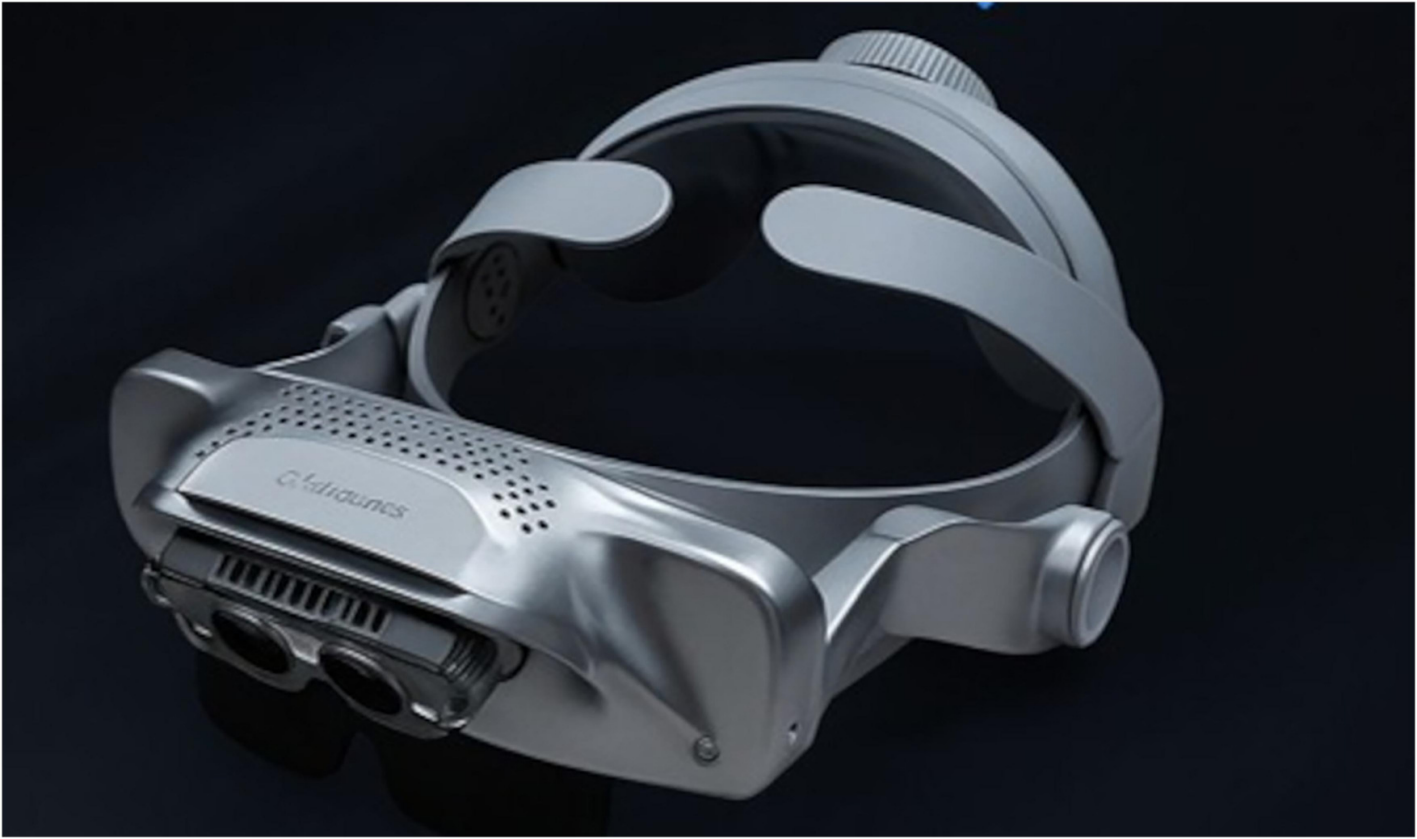
Digital magnification system (x3 to x6 or x4 to x8, bewelltech®, magnificent vision, Nankin, China).

## 3 Bioactive framework

The bioactive dental concept identifies the optimal bioactive materials for each clinical case. The bioactive option is preferred when choosing between traditional composite resin and a bioactive material with similar properties and limitations. Sauro et al. (22) detail the current attributes and constraints of “bioactive” materials. Whenever feasible, material selection should be guided by functional properties that significantly influence clinical outcomes, such as biocompatibility, antibacterial efficacy, biofilm modulation, remineralisation potential, and overall biofunctionality (22). Moreover, a systematic review and meta-analysis by Albelasy et al. (23) examined clinical evidence comparing ion-releasing dental restorative materials (such as glass ionomer cements (GICs and compomers) with conventional resin composite restorations regarding secondary caries occurrence and marginal adaptation. The review included randomised clinical trials but noted a high risk of bias in over 60% of the studies, with only three reporting low risk of bias. Performance bias was prevalent, partly due to the distinguishable nature of the materials used. The quality of evidence (GRADE assessment) was rated as low for both secondary caries incidence and marginal adaptation outcomes, weakening confidence in the effect estimates. No significant difference was found between ion-releasing and resin composite materials in secondary caries occurrence, implying that ion release capability alone does not reduce secondary caries risk. However, short study follow-up periods and an overall low number of caries events limited the ability to detect long-term material performance. The review also emphasised that new ion-releasing materials emerging in recent years lack robust clinical data and require further high-quality investigation. This article, also, discussed how resin composites with bioactive glass (BG) release ions and precipitate hydroxyapatite, offering benefits in dental caries management. Ion release, particularly calcium, phosphate, and fluoride, helps prevent demineralisation and promote remineralisation of enamel and dentine. Ion release profiles depend on pH: acidic conditions (pH 4.0) encourage ion release, while artificial saliva (pH 6.4) supports apatite precipitation, which may seal gaps and reduce leakage at the dentin-composite interface. SEM and FTIR confirmed apatite formation. Previous research shows BG composites reduce bacterial penetration and secondary caries, and some commercial materials like “alkasite” form apatite even in acidic environments, indicating potential for caries prevention (24–26). Although clinical trials are lacking, *in vitro* evidence shows sustained ion release, surface apatite formation, pH increases, and bioactivity supporting remineralisation. Long-term protection may result from prolonged ion release and surface activity. Mechanical factors like mastication or brushing can extend ion release by exposing unreacted BG fillers. These materials alter the local environment to reduce erosion and promote remineralisation, suggesting clinical effectiveness (27, 28).

## 4 Lesion detection technologies

The use of non-invasive and micro-invasive preventive therapies is influenced by the detection technologies used. Some can offer critical information about the presence or absence of cavitation, caries activity (fluorescence, bio-photonic signalling) and the accessibility (pictures magnified) of the lesion, meaning the real possibilities to clean the suspected area and then control the biofilm long term. The use of those devices that combine different magnification options, pictures or videos recording and photonic signals like infrared or fluorescence is advised in combination for the greatest accuracy especially for the earliest lesions (Figures 1–3) (6, 29, 30). To avoid misunderstanding like false positive signals and over-treatment risk by the clinician, progressive learning is recommended. Fluorescent cameras typically demonstrate average sensitivity and specificity values of approximately 0.93 and 0.63, respectively. However, selecting instruments based on suitability in relation to the following criteria is advisable.

### 4.1 Main clinical factors affecting intervention thresholds for all detection tools

A carious lesion detection device should enable the clinician to assess three essential clinical parameters: cavitation, lesion activity, and cleanability. First, **cavitation**: the device should determine whether the surface is cavitated, ideally under magnification, to guide the selection of an appropriate management strategy ranging from non-invasive to invasive interventions. Second, **lesion activity**: it should help establish whether the lesion is active or arrested. This distinction is critical in deciding whether therapeutic measures, including the use of ion-releasing biomaterials, are indicated. Third, **cleanability**: the device should assist in evaluating whether the lesion is accessible to effective plaque control. This assessment includes consideration of cavitation dimensions, depth, size, anatomical location (particularly proximal surfaces), and overall morphology. Together, these three parameters provide a structured and biologically oriented framework for clinical decision-making.

### 4.2 Additional useful clinical carious lesion detection criteria include

Diagnostic tools should be appropriate for both pre-operative and intra-operative stages of care. They should allow high-quality image and video recording to facilitate documentation, communication, and longitudinal monitoring. Adjustable magnification is essential to meet varying clinical requirements and to enhance precision during diagnosis and treatment. Importantly, these technologies should improve the detection and monitoring, particularly in hard-to-access proximal areas.

## 5 Levels of operative interventions

The specificity and sensitivity of diagnostic tools and their technical characteristics (magnification levels, photonic signals) make it possible to define three levels of intervention: Non-invasive (NI), micro-invasive (McI), invasive treatment (I) (Table 1). For each level of intervention, expert-driven flow chart were proposed to make reading smoother and clearer, but they are not yet derived from consensus statements. The Supplementary Tables S9, S10 contain the detailed references for each product.

**Table 1:** Levels of operative interventions.

Levels of intervention	Descriptions
NI, Non-invasive interventions.	That include those administered by patients at home, under advice and guidance from members of the oral healthcare team: biofilm control, diet control and topical remineralising agents, without conditioning [for example, toothbrushing with fluoride toothpaste, casein phosphopeptide-amorphous calcium phosphate (CPP-ACP) agent application, specific fluoride mouth rinses etc.] and all professional preventive techniques, at office, aiming to remineralise (Supplementary Table S9–S11) like SDF (multifunctional use) (21) and dopped fluoride varnish applications with Bioglass or surface pre-reacted filling (S-PRG filler) (31).
McI, micro invasive interventions.	That include etching, chemically conditioning, coating and so microscopically altering the susceptible tooth surfaces, which encompass also micro-abrasion, sealants, resin infiltration or self-peptide applications (21), in case of little cavitated initial caries lesions.
I, Invasive restorative interventions included moderate and extensive lesions.	Materials products are summarised in Supplementary Table S10. That include all extensive lesion treatments and “Slot or tunnel” preparations which preserved the marginal crest.
Vital pulp therapy (VTP).	Class I [traumatic/iatrogenic] or class II [carious], partial and full pulpotomies, a short description was developed in Chapter 10.

## 6 Non-invasive and micro-invasive treatments for proximal, smooth and occlusal surface initial lesions depends on the CRsA and caries activity

That concerns mainly the initial non-cavitated lesion or with tiny cavitation, only visible with magnification. Non-invasive products at home and professional are resumed in Supplementary Tables S9, S10.

### 6.1 Proximal and smooth surfaces

The main professional treatment modalities include the application of silver diamine fluoride (SDF) (32–34), resin infiltration (RI) (35), and self-assembling peptide therapy (36, 37) (Figure 5). In addition, micro-abrasion of smooth surfaces (performed using air abrasion, sonic inserts, or chemical agents such as 6% hydrochloric acid (Opalustre®^,^ UltraDent, USA), can be combined with resin infiltration for the management of specific types of white spot lesions. SDF application offers multiple clinical benefits, as previously documented (38–41). These three treatments are intended exclusively for non-cavitated lesions, although their applicability depends on the level of magnification used during examination. SDF is generally preferred for patients at high caries risk (32, 33, 39). RI involves infiltrating a demineralized, non-cavitated lesion with a hydrophobic resin. Successful application requires a clean tooth surface free of visible cavitation. All three treatments, RI, SDF, and self-assembling peptide therapy, are suitable for proximal or smooth surfaces, including orthodontic white spot lesions, hypoplastic or fluorotic defects, other white spot lesions, and root surfaces. Evidence demonstrates that RI effectively arrests the progression of non-cavitated caries, although it does not provide bioactive properties (35). Cleaning and accessing proximal surfaces remain challenging. Tools such as orthodontic separators, plastic wedges, or ivory separators can facilitate proper access and assessment, guiding the choice between RI, SDF, or self-peptide application (Table 2, Supplementary Tables S9, S10).

**Table 2:** Optional treatments and materials for proximal and smooth surface initial lesions.

Caries risk/susceptibility assessment (CRsA)	High CRsA and active caries	Low CRsA
Proximal or smooth surface initial lesion supposed non cavitated.	Resin infiltrant with or without micro abrasion, Silver Diamine Fluoride (SDF), Self-peptide P11-4 (Figure 5).	Fluoride varnish application 3,4x per years or new fluoride varnish with bioglass.
Proximal or smooth surface initial lesion with suspicious small cavitation.	Resin infiltrant with or without micro abrasion, Silver Diamine Fluoride (SDF), Self-peptide P11- 4 + GIC or composite patch if caries reachable.	Resin infiltrant with or without microabrasion, Silver Diamine Fluoride + GIC or composite patch if caries reachable.
**Bioactivity and proof levels**: SDF has remineralisation and anti-bacterial properties. Proof levels: caries in children L1a, caries in older adults L1b-2. For the self-peptide which is supposed to creates an amino acid scaffold which promotes remineralisation the proof levels are L1b and L5 for laboratory data (42, 43). Concerning the resin infiltrant which has no bioactive activity that remains useful in arresting the proximal caries process: Proof levels: L1a and L2, (44) L5 (laboratory data) (35).		
**Clinical comments**: For patients with a high risk of caries, SDF application is recommended twice per year (39, 41). An Ivory Tooth separator could be mandatory to better access the proximal lesion. However, evidence-based dentistry does not clearly specify a preference among these three materials and constraints due to the CRsA and does not give a clear recommendation in case of a very small cavity visible thanks to the huge magnification used, which is nearly inaccessible. Issues remain concerning the self-peptide with narrow clinical applications and some studies suggest that the benefits of self-peptide are significantly enhanced when used alongside adjunctive agents such as fluoride varnish or CPP-ACPF, implying that its standalone efficacy may be limited. There is also evidence of reduced mechanical stability over time; one study reported a decline in specific properties after prolonged water storage (six months), raising concerns regarding the long-term durability of the remineralised enamel (45, 46).		

### 6.2 Occlusal lesions

**Figure 4:**
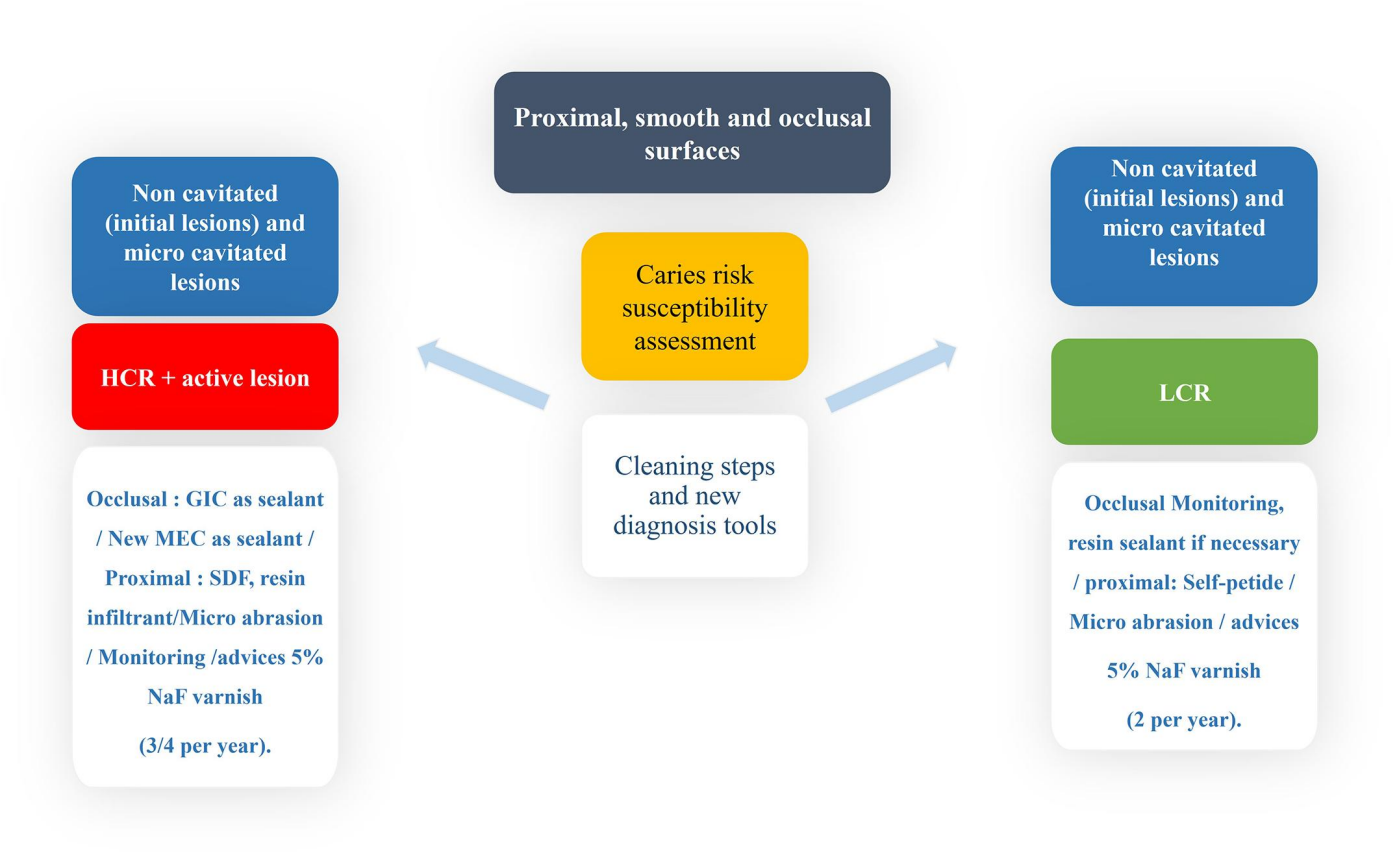
Expert-driven flow chart of non-invasive and micro-invasive treatment options for non-cavitated, initial proximal, smooth surface and occlusal lesions.

Sealants are regarded as one of the most effective therapeutic approaches for caries prevention among the various treatment options available for managing early occlusal carious lesions. This includes preventive fissure sealants (applied to healthy tooth surfaces in high-risk or susceptible patients) (40), therapeutic fissure sealants (applied to early demineralised surfaces without visible cavitation), or sealant restorations (where early, limited cavitation is visible under high magnification also known as preventive resin restorations). The treatment flowchart for occlusal surfaces is shown in Figure 4, which depends on the patient's caries risk or susceptibility and the accuracy of cavitation diagnosis. The patient's caries activity can be assessed using devices that produce fluorescence signals, although evidence of their efficacy is still under discussion (Table 3, Figure 6).

**Figure 5:**
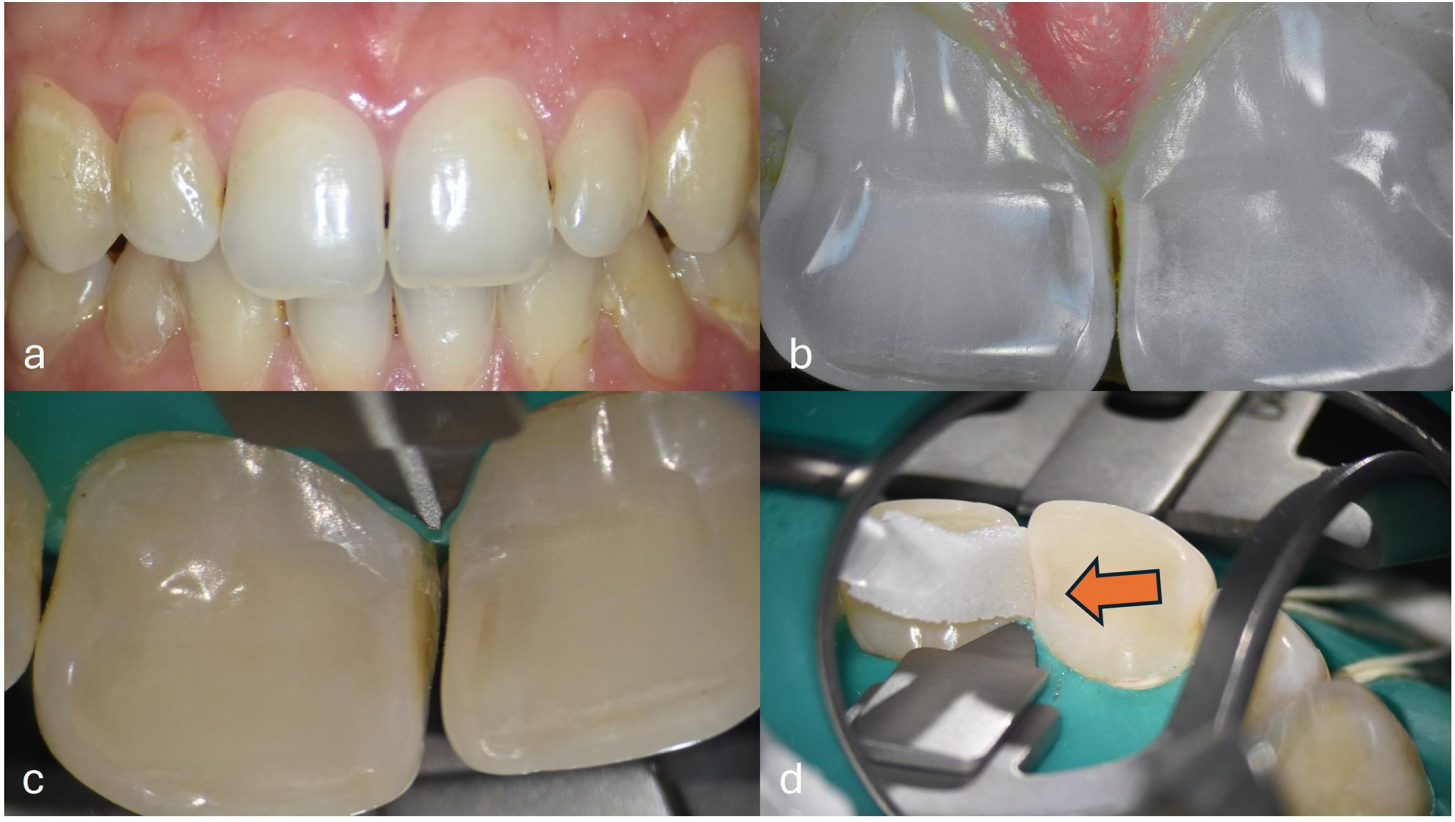
Non-invasive proximal caries ICDAS 2/3 treatment without cavitation with self-peptide solution. **(a)** Frontal view with microscope **(b)** Intraoral camera C50® (Acteon, France), mode boosted daylight view revealing the proximal white spot **(c)** Proximal view after cleaning with erythritol powder and teeth separated with “Teeth Ivory separator”, with no cavitation visible **(d)** Application of the self-peptide solution (soaked sponge, duration 5mn) (Curodont Repair®, vVardis, orange arrow).

**Figure 6:**
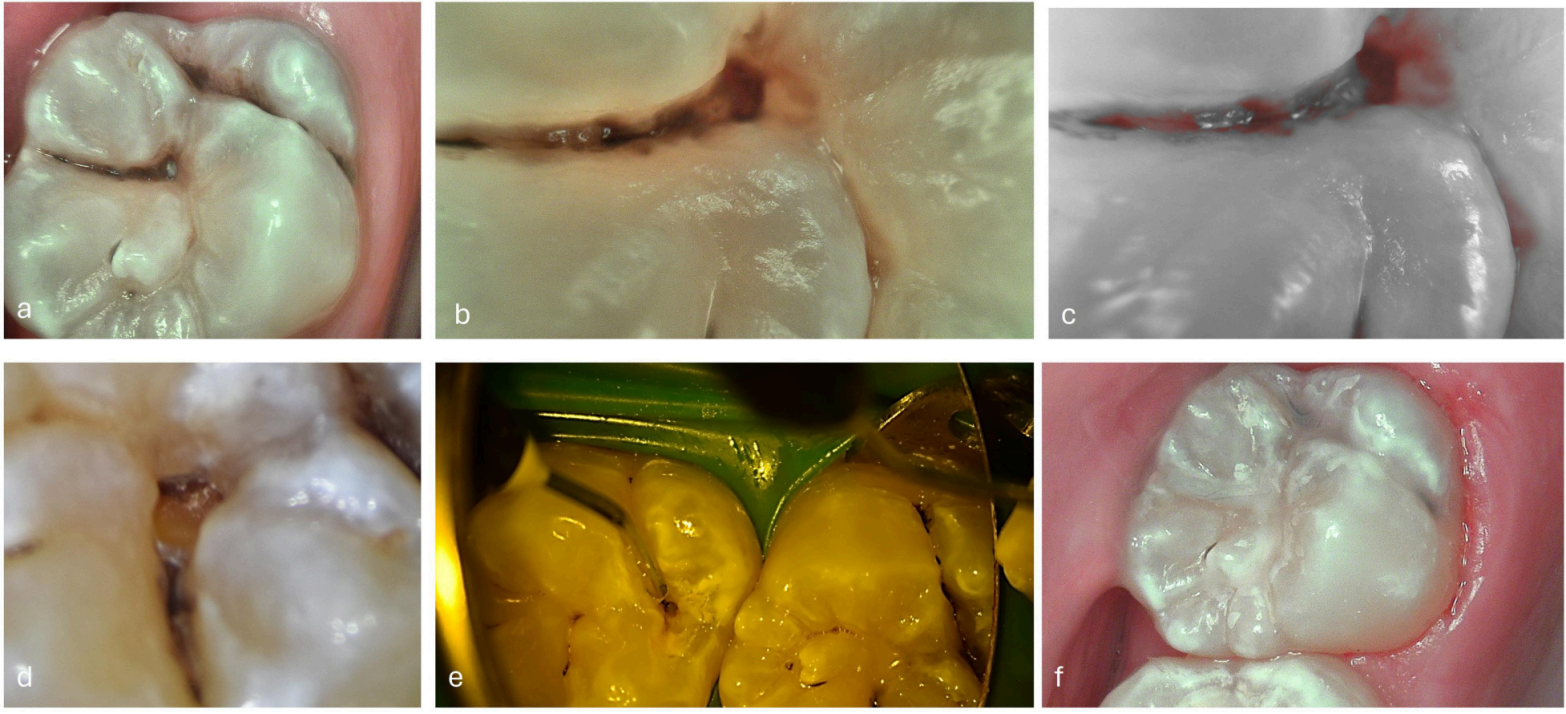
Occlusal fissure pattern in a high caries risk/susceptible patient-sealant restoration. **(a)** Daylight view (intraoral camera C50®, boosted mode, Acteon, France) with suspicious cavitated lesion and coloured fissures **(b)** Macro view in caries mode of the intraoral camera C50® **(c)** Red fluorescence signal revealing fluorescence deposits (Intraoral camera, caries mode, C50®) **(d)** Details of the ongoing occlusal preparation (Intraoral camera C50®) **(e)** Presto® injection **(f)** Occlusal view of mineral enriched composites (Presto®, Pulpdent, USA) as sealant.

**Table 3:** Optional treatments and materials for occlusal initial lesions.

Caries risk/susceptibility assessment (CRsA)	High CRsA and active caries	Low CRsA
Occlusal fissure with complex shape, non-cavitated.	If dental dam placement not feasible: GIC with finger press technique. Combination with SDF is optional. Mineral-enriched composite if moisture control adequate (47).	All resin-based sealants (if moisture control achieved, ideally with dental dam, etching and adhesive system mandatory) if occlusal fissure with complex shape. Mineral-enriched composite can be used too.
Occlusal lesion with small cavitation.	Mineral-enriched composite: only if dental dam application is feasible.	All resin-based sealants or composite flow (if application of dental dam feasible, etching, and adhesive system mandatory). Mineral-enriched composite can be used too if dental dam application is feasible.
**Bioactivity and proofs levels**: Dental sealants are supported by high-level evidence (Level 1) for preventing caries in children and adolescents. Comparisons of specific materials rely on Level 2 evidence from RCTs, with some methodological limitations noted (48, 49). EBD remains cautious about the use of MEC versus traditional sealants, even if the first results show very encouraging results (Level 1–2, Systematic/RCTs for Activa®) (47, 50). Currently, despite some case reports, it does not seem reasonable to recommend self-peptide and resin infiltration for the treatment of occlusal lesions.		
**Clinical comments**: The evidence is insufficient to determine which sealant type is superior clearly. A 76% reduction in caries is possible, supported by moderate evidence and strong recommendations (51, 52). Clinicians should consider caries risk and dental dam use when choosing between GIC and mineral-enriched resin composites. Mineral-enriched composites alter the traditional resin composite approach by offering similar performance (53). Associating the SDF with the GICs appears promising but remains not yet clearly recommended by the EBD (54).		

## 7 Therapeutic options for excavation and tissue conditioning for invasive caries lesions (moderate and extensive)

### 7.1 Common recommendations

Prior to the excavation steps, the tooth should be thoroughly cleaned using airflow with soft powders to remove biofilm and debris while preserving the integrity of the hard tissues (8).

Where available, magnification and photonic technologies, such as fluorescence or infrared, should be employed to reassess the lesion and evaluate caries activity. The Peripheral Seal Concept should be applied when residual affected dentin is intentionally left, targeting peripheral excavation to sound hard dentin (19). This approach can be integrated with the selective excavation concept to maximize tissue preservation. Preservation of the gingival enamel margin is recommended whenever possible. Polymer burs provide an optimal balance between caries removal effectiveness (CRE) and minimal invasiveness potential (MIP), allowing effective removal of soft carious tissue while minimizing damage to surrounding tooth structure (20). For moderate lesions (ICCMS score 3–4), excavation may proceed to firm dentin. For extensive lesions (ICCMS score 5–6), excavation should be limited to leathery dentin, using IRB as dentine substitute (20).

### 7.2 Various optional cavity conditioning methods available before applying the adhesive system

Air abrasion using bioglass 45S5 can create a therapeutic, “bioactive” smear layer that protects the bonded interface while maintaining adhesion performance (22, 55, 56). An additional application of chlorhexidine (CHX 2%, 1 min, no rinsing) has been suggested for its antibacterial properties and inhibitory effect on matrix metalloproteinases (MMPs); however, its routine use remains debated (57), Photo-activated disinfection (PAD) with photoactive compounds, such as Tolonium chloride (Dentfotex®, USA), followed by light activation and rinsing, produces oxygen-based free radicals. The effectiveness of PAD is limited, however, as these reactive oxygen species diffuse only approximately 100 nm and have a very short half-life (58, 59).

Sodium hypochlorite (NaOCl) can also be applied before restorative procedures; for example, 6% NaOCl for 15 s followed by rinsing. The hybrid layer remains intact after deproteinization, even with 10% NaOCl gel, provided the application time does not exceed 30 s (42). Chemo-mechanical caries removal (CMCR) agents, such as enzyme gels like Papacaries®, may be used either before self-etching or after total-etching techniques, offering additional antibacterial effects (60, 61). Dentin surface treatment with 37% phosphoric acid for 5 s has no adverse effect on the bonding of resin-modified glass ionomer cement (RMGIC) compared with treatment with polyacrylic acid for 10 s (4, 17, 55). Moreover, the combined use of silver diamine fluoride (SDF) with CMCR methods, such as papain-based enzyme gels, may further enhance antibacterial efficacy (62).

### 7.3 Adhesive systems options including antiseptic molecules or specific ions

In addition, adhesive systems containing 0.2% chlorhexidine **(**CHX) (63), or antibacterial monomers such as 12-methacryloyloxydodecylpyridinium bromide (MDPB) provide targeted antibacterial activity against key cariogenic bacteria, including *Streptococcus mutans*, *Lactobacillus casei*, and *Actinomyces naeslundii*. These adhesives can help disinfect cavities that retain residual bacteria after caries removal (64, 65). Emerging universal adhesive systems incorporating bioactive or antimicrobial agents, such as zinc oxide, copper nanoparticles, titanium dioxide (TiO₂), cerium dioxide (CeO₂), L-arginine, loaded mesoporous silica nanoparticles, or theobromine, demonstrate promising results in enhancing the hybrid layer and improving the interface with caries-affected dentin. These effects have been observed in both *in vitro* and *in vivo* studies (66–69).

### 7.4 Common limiting clinical factors

Common limiting factors include the inherent challenges in accurately diagnosing non-cavitated and micro-cavitated lesions, as well as limited accessibility to the deepest portions of fissures where residual biofilm may persist. Assessing caries activity with precision remains difficult, and the actual effectiveness of ions released by bioactive materials (as described in the ABRAM framework) at the dentin interface is not fully established. Additional limitations involve long-term monitoring of structural changes and the stability of the hybrid layer in contact with individualized risk–benefit (IRB), guided interventions, as well as the practical realities of dentin remineralization, particularly considering residual water within collagen fibres (56, 70). Evaluating the remineralization potential at the dentin interface, whether with glass ionomer cements (GIC), high-viscosity GICs (HVGIC), newer modified experimental cements (MEC), or doped bioactive materials, remains challenging in *in vivo* clinical situations (22, 55, 71).

## 8 Treatment for proximal and occlusal moderate and extensive lesions depends on the CRsA and caries activity

**Figure 7:**
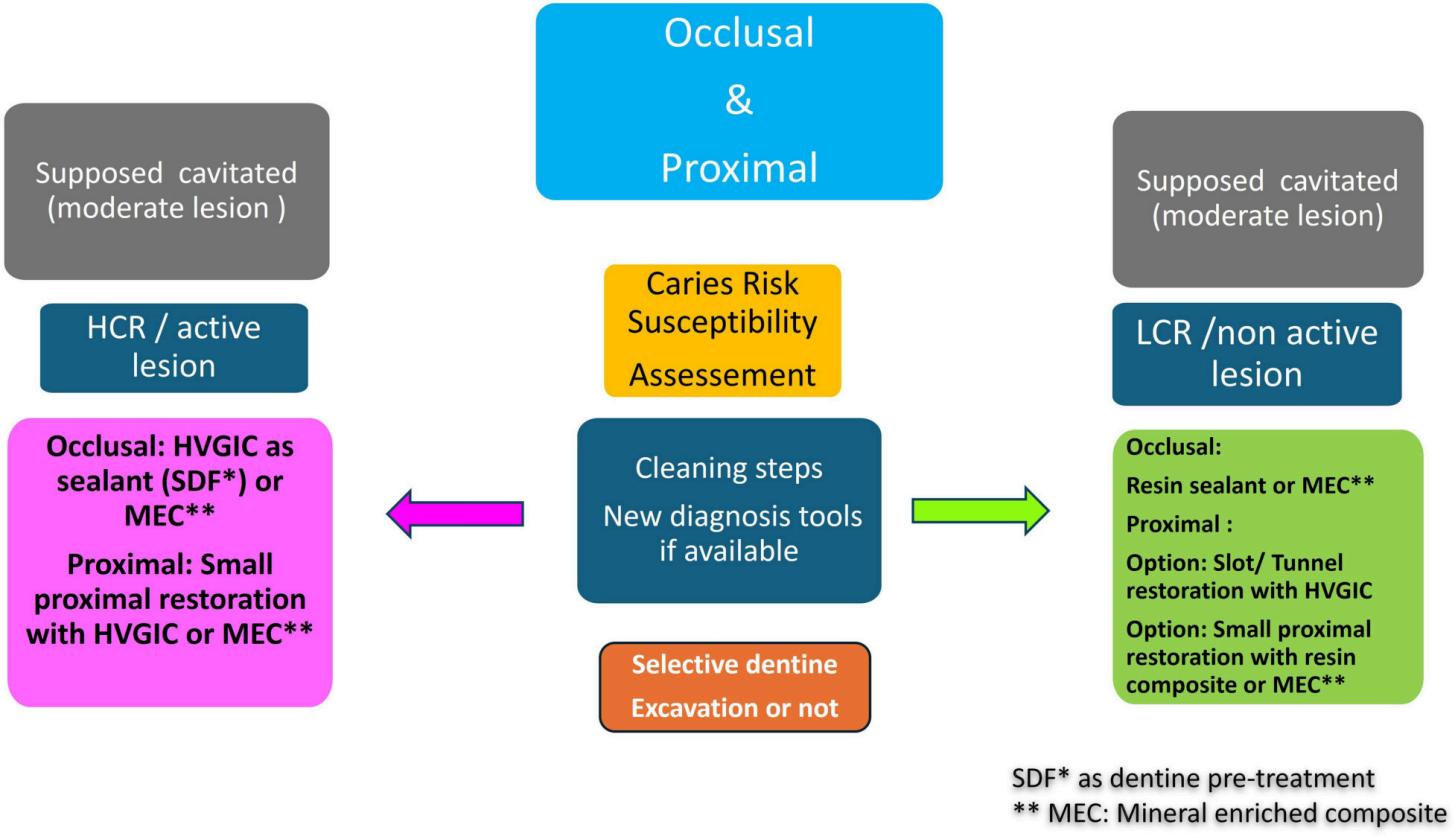
Expert-driven flow chart of microinvasive treatment options for moderate occlusal and proximal lesions. (MEC: mineral enriched composite, SDF: silver diamine fluoride, HVGIC: high viscosity glass ionomer cement).

Treatment for proximal and occlusal moderate and extensive lesions are designed as invasive treatments (Figures 7, 10). Note that SDF can be combined with GIC as dentine conditioning to reduce the caries process in case of HCR and active caries. Available products are described in Supplementary Tables S9, S10.

### 8.1 Moderate occlusal carious lesions

For moderate occlusal lesions, clinical difficulties are reduced to 3 points as the enamel border often surrounds the entire cavity preparation: 1) CRsA: HCR for High caries risk or LCR for Low caries risk. 2) Carious lesion activity and diagnosis. 3) Excavation can be extended to the firm or leathery dentine layer (20) (Figure 7).

### 8.2 Moderate proximal lesion with bondable enamel

**Figure 8:**
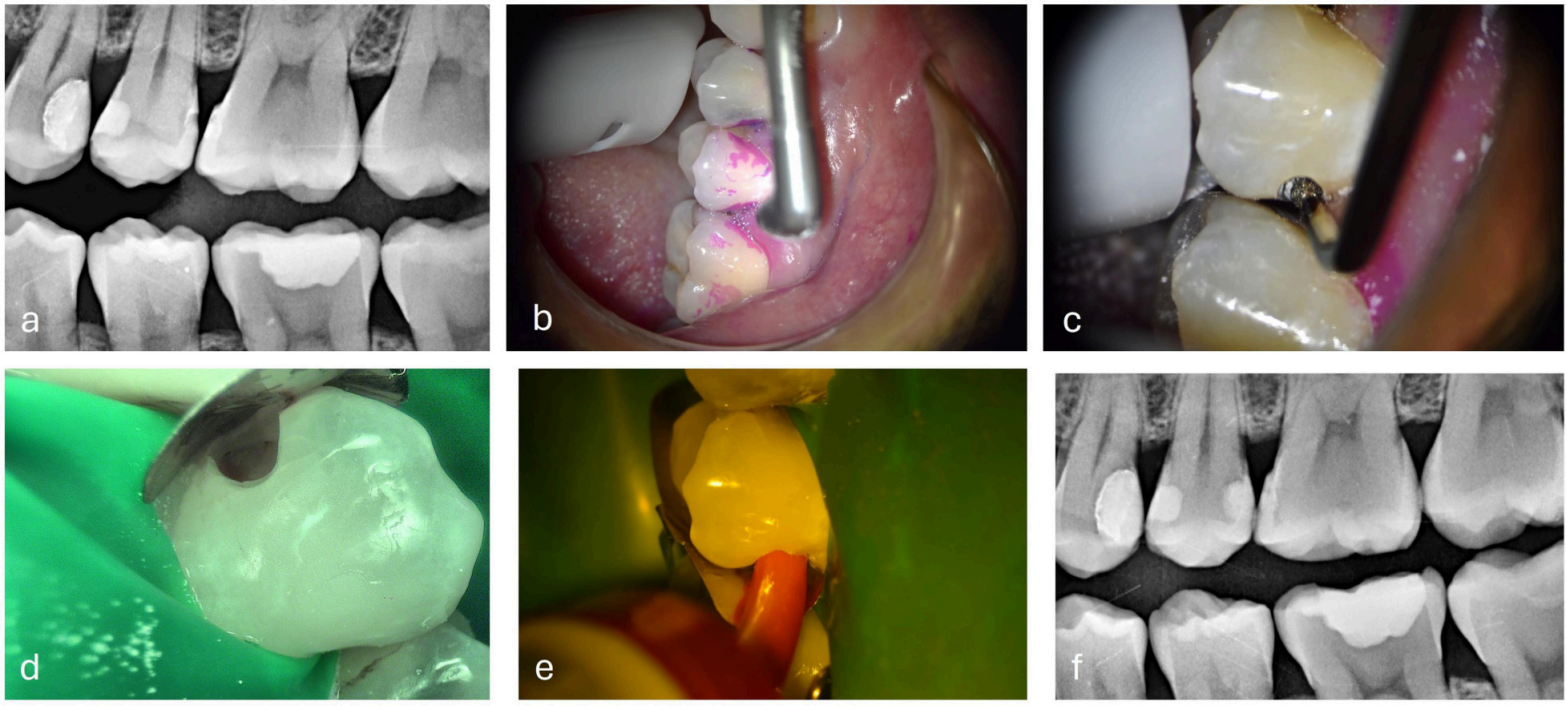
Slot cavity on a distal carious lesion of an second upper premolar. **(a)** Bitewing x-rays **(b)** Cleaning step with airflow, EMS dye plaque **(c)** US diamond insert half-round, (NSK®, Japan) **(d)** Slot preparation with metal matrix setting, intraoral C50® camera view (Acteon, France) **(e)** HVGIC LC injection (Riva® SDI, Australia), **(f)** Bitewing x-rays control.

**Figure 9:**
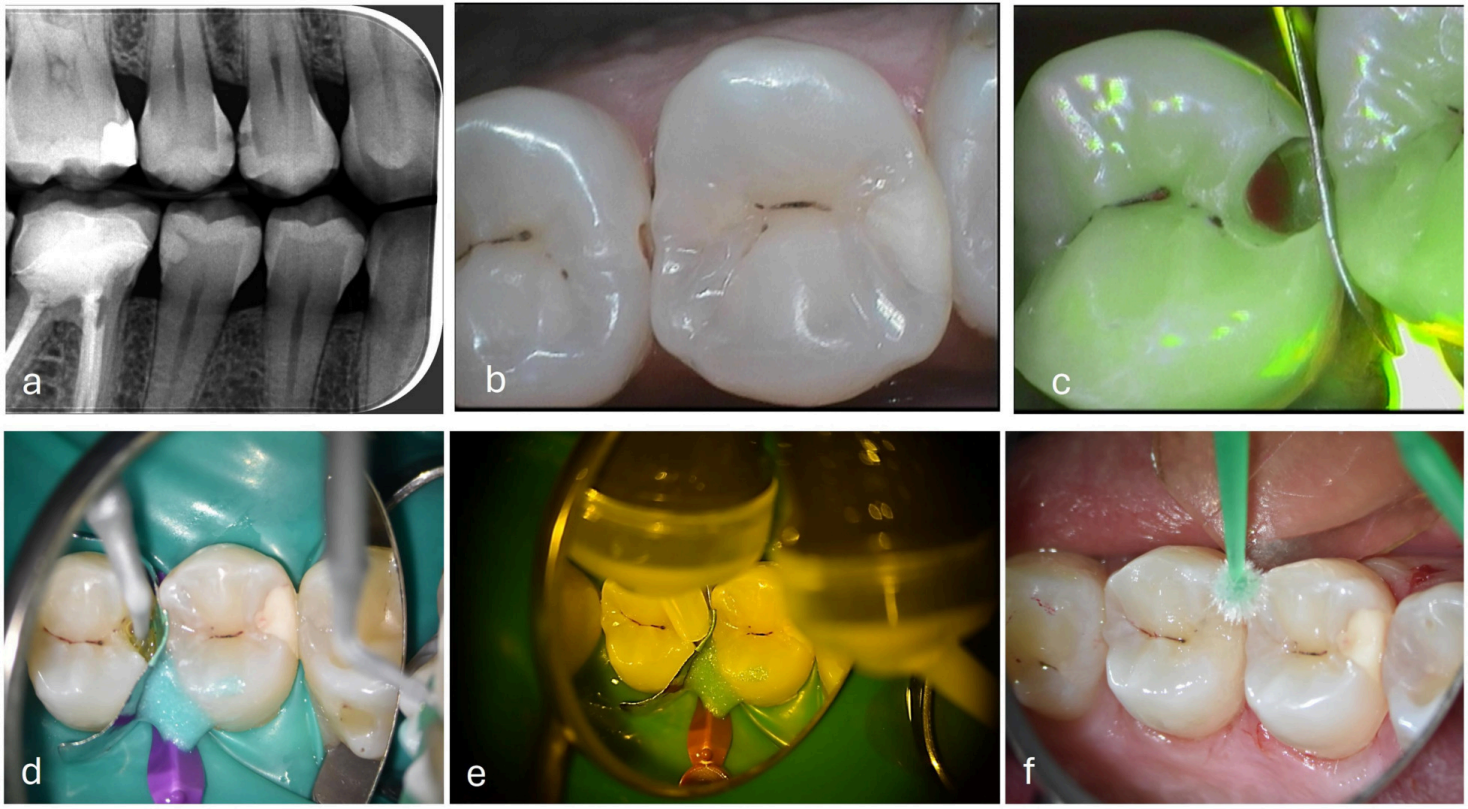
Distal Cl II moderate preparation (tooth 14) on HCR patient with cention® (ivoclar, Liechtenstein). **(a)** Bitewing x-rays **(b)** Visible cavitation after air flow cleaning **(c)** Fluorescence mode (Soprolife® view, Acteo, France) **(d)** Cention®+ primer **(e)** Cention® injection **(f)** Cention® + fluorid varnish.

For proximal lesions with bondable enamel, clinical difficulties are based to 4 points: 1) CRsA: HCR for High caries risk or LCR for Low caries risk. 2) Carious lesion activity and diagnosis. 3) Excavation can be extended to the firm or leathery dentine layer (20). Presence or not of bondable enamel in gingival margins, but in case of moderate many lesions, many times the enamel cervical border still exists. (see above, invasive lesion with no bondable enamel). (Figures 8, 9) (Table 5).

**Table 4:** Optional treatments and materials for moderate occlusal lesions.

High CRsA and active caries	Low CRsA options for caries activity
IRB are preferred as temporary materials except Alkasite composite. Flow conventional GIC: high fluoride ion releasing for a temporary restoration, conventional GIC, High viscosity GI (self or light cure) (72).	IRB as dentine substitute if active caries. Option 1: If RMGIC: apply “dentine conditioner” (optional), rinse than inject RMGIC as dentine substitute.
Optional: SDF can be combined with GIC as dentine conditioning to reduce the caries process.	Option 2: If RMGIC-based composite, (Mineral-enriched composite) can be used with “Universal adhesive” in etch-rinse (5s selective etching) may contribute to maintain the bonding performance (17).
	Option 3: If HVGIC (self or light cure): applied with “dentine conditioner” (optional), rinse than injected HVGIC.
	Option 4: If inactive caries: All options available.
**Bioactivity and proofs levels**: According to the Oxford classification, the level of evidence for high-viscosity GIC as a dentine substitute is moderate (Level 3–4), supported by multiple RCTs and cohort studies, though long-term comparative data are limited (54, 73, 74).	
**Clinical comments**: Few materials besides calcium silicate cements, mainly used for vital pulp therapies (L2), can form a hydroxyapatite-like structure. Most materials infiltrate the hybrid layer and began to outperform conventional composite. Current level of evidence for Alkasite material as a dental restorative material is moderate at best, based on one-year randomized clinical trial data showing comparable performance to composite restorations, supported by *in vitro* mechanical studies, while bioactivity findings remain preliminary and further clinical trials are awaited (24, 75). New generations of dopped GIC with zinc ions was developed by GC (Japan) with promising results (76). However, the product is less available on the dental market and not available in Europe. Biomaterials like Giomer® positioning at the boundary between GIC and resin composite or specific coating containing S-PRG filler seem to prevent caries while supporting enamel remineralization, reduce dentin hypersensitivity with immediate and lasting relief but long-term clinical trials are required to confirm durability and therapeutic effects (77–79).	

**Table 5:** Optional treatments and materials for proximal moderate lesions.

High CRsA and active caries	Low CRsA options for caries activity
See therapeutic options for occlusal lesions for HCR patients (Table 4). Avoid sophisticated cavitary preparations and prefer classic proximal preparations. Optional: SDF can be combined with GIC as dentine conditioning to reduce the caries process.	Option 1: If a slot or a tunnel preparation (Figure 8), the use of IRB for active and non-active carious lesions remains mandatory, and it is preferred HVGIC LC or RMGIC as it is easier to use due to the longer setting time and is easiest to remove in case of overflow. The occlusal increment of the tunnel restoration is preferably covered with a resin composite after adhesive procedures. Be careful in case of SDF application as dark shadows could appear through the marginal crest.
	Option 2: If conventional preparations, all techniques are possible in case of non-active carious lesions and prefer IRB as a dentine substitute in case of active caries.
	Option 3: Alkasite composite (Figure 9) or mineral enriched self-adhesive composite or dual MEC. If no bondable enamel, see proximal extensive caries lesion.
**Bioactivity and proofs levels**: Because studies focused specifically on slot or tunnel (80, 81) preparations are scarce, their best-supported grade is low (Level 3–5). Evidence for traditional Class II composite restorations is stronger overall, with multiple RCTs and systematic reviews (Level 1–2) evaluating longevity and failure modes, but not isolating the “slot” design as a subgroup (82–84).	
**Clinical comments**: Although slot or tunnel preparation may require additional time, preserving the marginal ridge remains critical for optimal outcomes. Achieving this objective necessitates using specialised diagnostic tools and preparation instruments (9, 85).	

## 9 Invasive treatment for proximal and occlusal extensive lesions depend of the CRsA and caries activity

**Figure 10:**
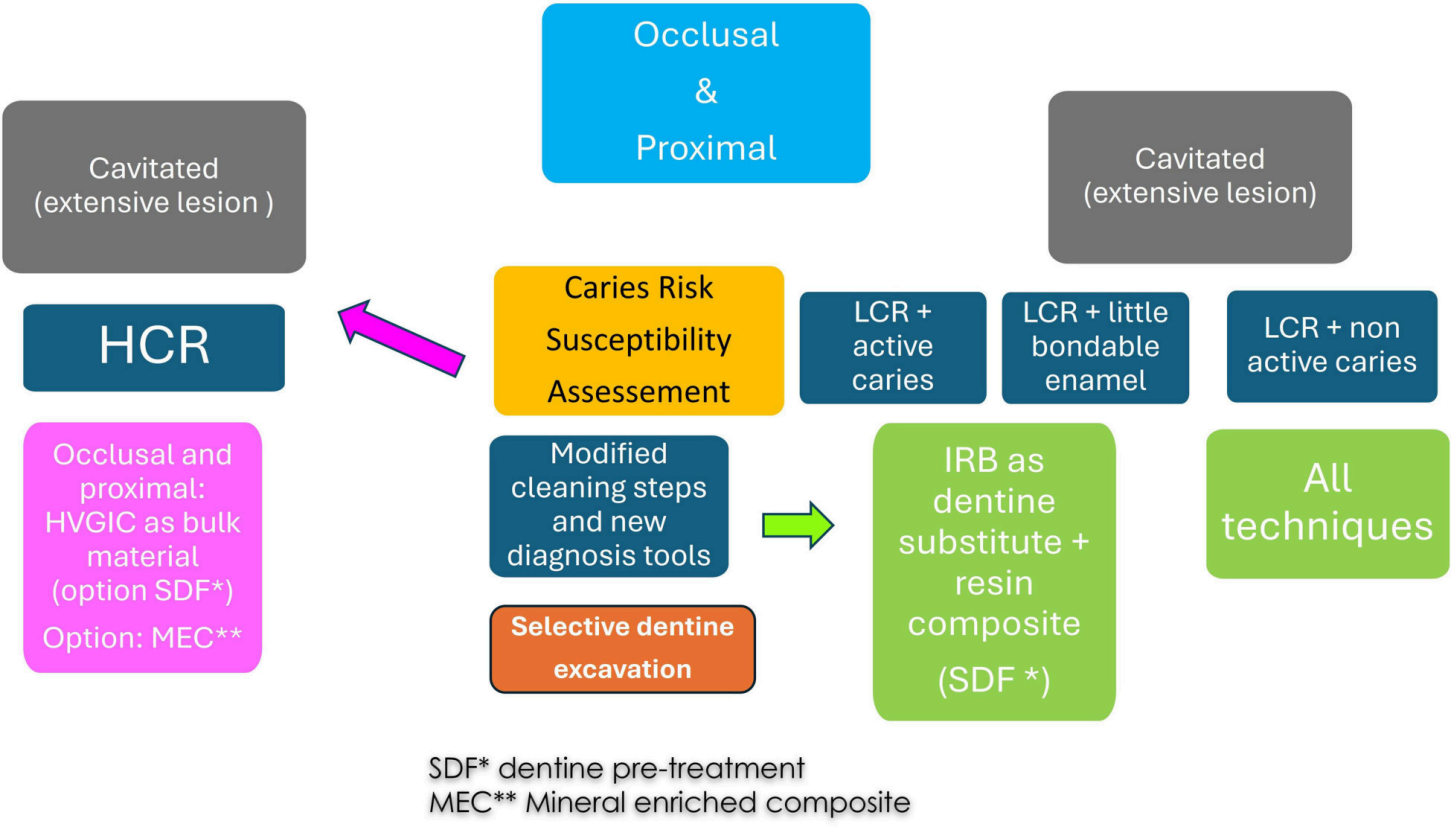
Expert-driven flow chart of invasive treatments options for extensive cavitated, occlusal and proximal lesions. (MEC: mineral enriched composite, SDF: silver diamine fluoride, HVGIC: high viscosity glass ionomer cement).

The treatment of these lesions seems to depend on the patient's risk of caries, the presence of active caries, and the absence of an enamel border in the cervical area (Figure 10). Available products are described in (Supplementary Tables S9, S10).

### 9.1 Extensive occlusal lesions

For extensive occlusal lesions, clinical management involves several key considerations. First, the patient's caries risk must be stratified, distinguishing between high caries risk (HCR) and low caries risk (LCR), as this informs the overall treatment approach. Second, accurate assessment of lesion activity is essential to guide decision-making. Third, excavation may be extended to the leathery dentin layer when clinically indicated (20). Finally, bondable enamel is typically present around the preparation to support adhesion. In situations where only soft dentin remains, restoration should rely exclusively on high-viscosity glass ionomer cement (HVGIC), allowing for appropriate clinical follow-up and monitoring of lesion progression (18) (Table 6).

**Table 6:** Optional treatments and materials for occlusal extensive lesions.

High CRsA	Low CRsA options for caries activity
HVGIC (combination is optional with SDF) or Calcium silicate base material as temporary materials (protected with GIC) in case of vital pulp therapy. Optional: SDF can be combined with GIC as dentine conditioning to reduce the caries process.	LCR + active lesion: IRB like HVGIC as dentine substitute. In case of a pulpal proximity consider applying a bioactive liner like calcium silicate cement. Optinal: application of SDF.
	Option 1: Alkasite composite (Figure 11).
	Option 2: Mineral enriched self-adhesive composite (86) or dual MEC.
	Option 3: If LCR + inactive lesion: all therapeutic options are available.
Bioactivity and proof levels: Consistent with moderate lesions. The levels of evidence of real bioactivity of these new materials are still low (L3–4), but mainly due to the lack of publications (24, 25).	
Clinical comments: New materials are changing the paradigm of material choice such as Stela® (SDI, Australia), which offers a new mode of polymerization (bottom-up self-cured) or new MEC bulk and dual cured and both seems very promising. Even if its bioactivity capacities are reduced, they must be, carefully, taken into account. The best choice between all these options is not given by the EBD (53, 85).	

### 9.2 Extensive proximal lesion with non-bondable enamel

**Figure 11:**
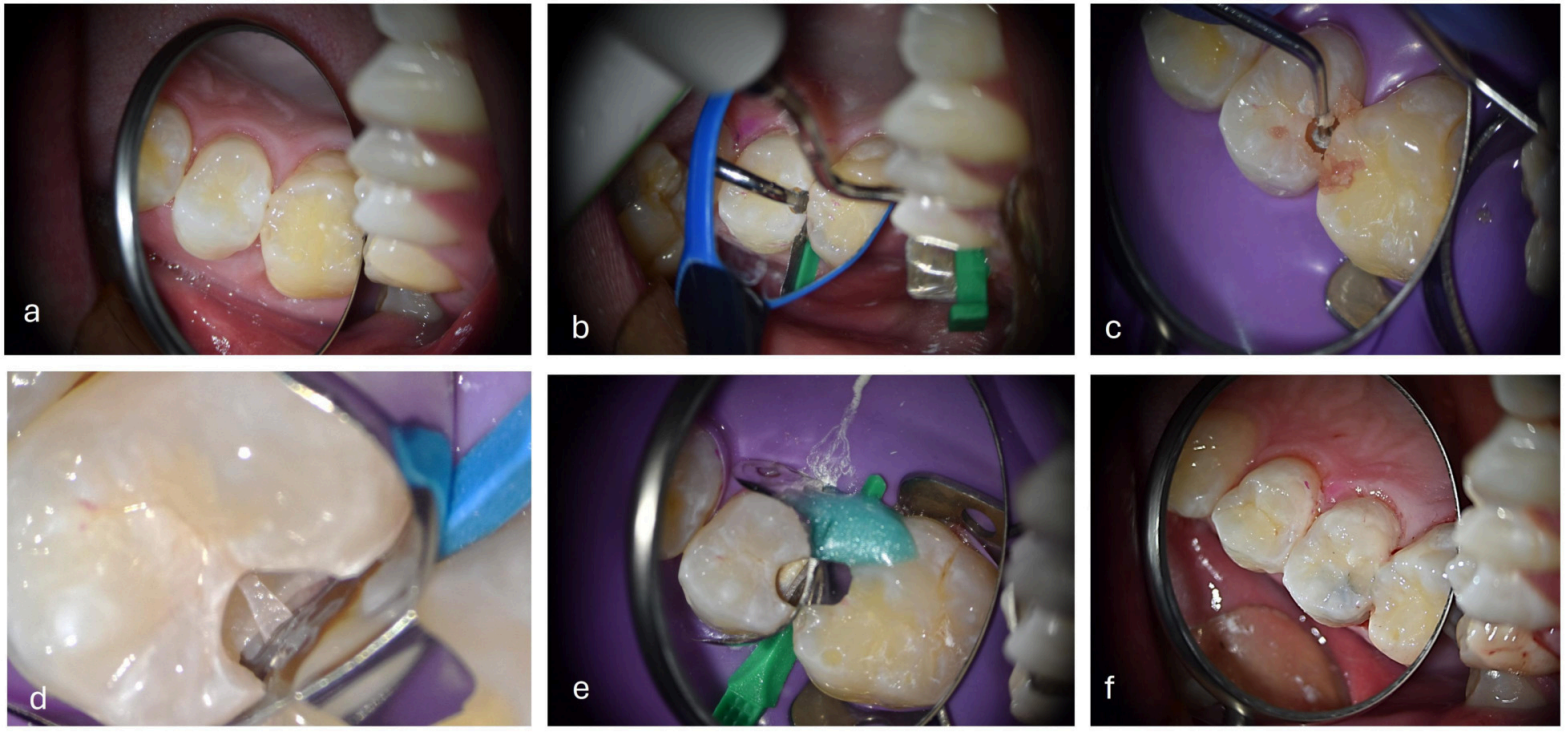
Second premolar with distal extensive proximal cavity. **(a)** Occlusal view **(b)** Semicircular diamond insert to shape the cavity **(c)** Special hand excavator to preserve the pulp complex **(d)** Partial cervical edge rupture **(e)** Modification of the dentine looking after SDF (Aquabond® 1 and 2 application, Riva®, SDI, Australia) **(f)** HVGIC LC obturation (Riva®, SDI, Australia).

For proximal extensive lesions, clinical difficulties are based to 4 points: 1) CRsA: HCR for high caries risk or LCR for low caries risk. 2) Carious lesion activity and diagnosis. 3) Excavation can be extended to the leathery dentine layer (49). 4) Absence of residual bondable enamel at the gingival margin: This finding indicates that the lesion should be classified as an active caries (Table 7) (Figure 11).

**Table 7:** Optional treatments and materials for proximal extensive lesions.

High CRsA	Low CRsA options for caries activity
HCR: Favor HVGIC (self or light cure) to support the cavity constraints or apply a calcium silicate based material in case of vital pulp therapy (Protected with GIC). Optional: SDF can be combined with GIC as dentine conditioning to reduce the caries process.	LCR: IRB as dentine substitute for both active or inactive caries (Figure 11).
	If active caries: Option 1: RMGIC or HVGIC (self or light cure) as dentine substitute, in open sandwich, + resin composite. SDF is optional as dentine conditioning if active caries.
	Option 2: Alkasite composite or mineral enriched self-adhesive composite.
	Option 3: Calcium silicate based material in case of vital pulp therapy (Protected with GIC).
	Option 4: if LCR + inactive lesion: all technics are available.
**Bioactivity and proof levels**: Consistent with moderate lesions. It is not possible to make an EBD hierarchy on the 4 options (41, 87, 88).	
**Clinical comments**: New mineral enriched self-adhesive composite are changing the paradigm of material choice, which offers a new mode of polymerization and seems very promising for deep marginal elevation (89). In the same spirit, new bulk-dual composite looks promising too, even if both their bioactivity capacities are reduced, they must be considered. A limitation of EBD is that it does not provide specific recommendations when bondable enamel levels are low in contrast of the Bioactive dental concept. Choosing between HVGIC, RMGIC, Alkasite composite or new mineral enriched composite as a dentine substitute or bulk materials is challenging, and EBD offers no clear guidance. The application of SDF in case of active caries before application of GIC seems promising (54).	

## 10 Vital pulp therapy (VTP)

VPTs [class I [traumatic/iatrogenic] or class II [carious]],and partial and full pulpotomies, are generally considered less controversial when supported by solid clinical evidence across various therapeutic contexts (Table 8). In direct pulp capping procedures, hydraulic silicate cements are recognized for their favourable biological and therapeutic properties, while resin-based materials offer greater facilities of use but lower biological efficacy (88, 90–92). The recent introduction of Biodentine® XP cartridges incorporates both powder components (tricalcium silicate, zirconium oxide, calcium oxide, calcium carbonate, iron oxides) and an aqueous solution consisting of calcium chloride, polycarboxylate, and purified water, resulting in enhanced material properties. Technical improvements have achieved a compressive strength of at least 150 MPa within 24 h, with a final strength ranging from 260 to 300 MPa, comparable to that of healthy dentin, as well as a faster and more reliable setting time. Successful clinical outcomes, however, rely not only on the material but also on meticulous procedural execution: use of magnification, accurate assessment of pulp vitality (for example, by observing the nature and rate of pulp bleeding), strict isolation and asepsis, careful coagulation control, gentle application of the capping material without excessive pressure, and proper sealing with a high-viscosity glass ionomer under certain situations but should not be applied as an open sandwich in proximal restorations. For further reference, material properties are summarized in the Supplementary Table S10 (88).

**Table 8:** Optional products for Vital Pulp Therapy.

**Hydraulic calcium silicate-based** (Tricalcium silicate (purified))	**MTA** (Tricalcium silicate, dicalcium silicate, tricalcium aluminate), **Resin Modified MTA**
**Bio-Interactive Properties:** These materials set through hydration and precipitation, with an alkaline reaction that promotes remineralisation, decreases MMP activity, and provides antibacterial benefits for caries-affected dentine.	**Bio-Interactive Properties**: Exhibits bio-interactive principles similar to calcium silicate-based materials, though with little reduced effectiveness.
**Biological Effects:** Collagen fibril degradation creates a porous structure, boosting Ca and carbonate ion penetration and enhancing mineralisation at the interface zone.	**Biological Effects**: Suitable for vital pulp therapy; facilitates dentine bridge like formation less than MTA and but offers user-friendly application.
**Drawbacks:** Slow setting time; suitable mainly as liners or temporary restorations with efficient biological effects. MTA®, can't be used as dentine substitute in contrast of the Biodentine® (only close sandwich for Biodentine®).	**Drawbacks**: Primarily utilized as a liner near the dental-pulp complex, but demonstrates fewer biological effects compared to Biodentine ®. Can't be used as dentine substitute.

## 11 Contemporary bioactive materials and future prospectives

Recent developments in bioactive dentistry have progressed beyond the traditional application of glass-ionomer cements and early ion-releasing composites. Contemporary ion-releasing “bioactive” materials now encompass nanocomposite-based systems, calcium-silicate cements, bioactive glass-enhanced composites, giomers, alkasite materials, and self-adhesive, dual-cure restorative systems endowed with bioactive properties (93). Recent reports underscore innovations such as nanotechnology-enhanced bioactive composites that augment strength, wear resistance, and antimicrobial activity, as well as intelligent, stimuli-responsive materials that adapt to pH variations within carious lesions. Moreover, advances in bioactive adhesive systems, fibre-reinforced composites, and ion-releasing restorative materials designed specifically for elderly populations exemplify ongoing improvements in biological compatibility and restoration durability (94). Indeed. These materials will actively interact with tooth tissues and the oral environment. These materials promote remineralisation, enhance interfacial stability, release ions such as Ca^2+^, PO₄^3−^, and F^−^, and exhibit antimicrobial or pH-modulating behaviour, thereby reducing secondary caries and improving long-term restoration survival. Recent reports highlight innovations such as nanotechnology-enhanced bioactive composites that improve strength, wear resistance, and antimicrobial activity, as well as smart, stimuli-responsive materials that adapt to pH fluctuations within carious lesions. Moreover, future trajectories include establishing standardised bioactivity testing protocols; enhancing long-term mechanical performance through novel calcium-phosphate fillers; developing antibacterial and self-healing composites; and integrating AI-assisted material selection, 3D printing, and bioprinting technologies to produce patient-specific bioactive restorations (22). These advancements signify a prominent shift towards biologically interactive, regenerative, and personalized restorative dentistry, aligning closely with the minimal-intervention philosophy articulated in our manuscript (95).

## 12 Conclusion

The findings of this work should be viewed as hypothesis-generating rather than definitive, since the study design did not aim to confirm clinical superiority using S3-level evidence-based guideline methodology. Models like the S3 framework, based on quality ratings for outcomes such as failure, tooth survival, postoperative hypersensitivity, secondary caries, and longevity, are helpful for standardising recommendations, but they can also be overly restrictive. By heavily weighting criteria like risk of bias, inconsistency, or imprecision, these systems may lead to conclusions that overlook promising approaches, including the use of liners or dentin substitutes in deep caries management. Our observations suggest that such rigid interpretations may not always reflect clinical reality. The same limitation applies to novel bioactive materials and technologies, such as recently developed chemically cured restorative systems or next-generation fluorinated varnishes. Although early results and mechanistic data are encouraging, these products often lack the volume of long-term randomised evidence needed for high-level grading, which should not be mistaken for a lack of promise or clinical value. Likewise, diagnostic innovations, such as fluorescent intraoral cameras, have demonstrated notable clinical benefits despite historically receiving only moderate levels of evidence, sometimes from reviewers who have never used the technology.

Our findings support the biological plausibility and clinical relevance of ion-releasing, bioactive restorative materials. The mechanistic roles of calcium, phosphate, and other ions (fluoride, zinc, magnesium, silanols) in fostering dentin remineralisation, hybrid layer stabilisation, enzymatic protection, pH buffering, and microbial modulation are well established. These mechanisms align with the clinical goals of minimally invasive dentistry and selective caries removal. However, while our results concur with these mechanisms, they do not serve as definitive proof that any particular material offers superior long-term clinical performance. Larger, longer-term randomised trials are necessary to test that hypothesis. This study also highlights a broader challenge: strict adherence to evidence-based dentistry (EBD) can unintentionally limit clinical options by excluding emerging techniques or materials simply because they lack sufficient high-level evidence. Many widely adopted clinical practices, such as behaviour management techniques like “Tell-Show-Do,” have long histories of practical success despite lower evidence ratings in systematic reviews. Classic examples outside dentistry, such as the untestable yet universally accepted benefit of parachutes, illustrate that not all interventions are amenable to RCT validation. For this reason, we advocate a balanced approach to clinical decision-making: one that remains evidence-aware, acknowledges methodological rigour, but also recognises the practical realities of dental care. Our study offers preliminary comparative observations that can guide future research and help clinicians understand the biological and functional rationale for bioactive materials. Nonetheless, confirming clinical superiority will require further controlled, long-term randomised clinical trials.

## Data Availability

The original contributions presented in the study are included in the article/Supplementary Material, further inquiries can be directed to the corresponding author/s.

## References

[1] Marovic D Par M Posavec K Marić I Štajdohar D Muradbegović A Long-Term assessment of contemporary Ion-releasing restorative dental materials. Materials. (2022) 15:4042. 10.3390/ma15124042 35744101 PMC9227571

[2] Vallittu PK Boccaccini AR Hupa L Watts DC. Bioactive dental materials—do they exist and what does bioactivity mean? Dent Mater. (2018) 34:693–4. 10.1016/j.dental.2018.03.001 29571660

[3] Hench LL. The story of bioglass®. J Mater Sci Mater Med. (2006) 17(11):967–78. 10.1007/s10856-006-0432-z 17122907

[4] Pires PM de Almeida Neves A Lukomska-Szymanska M Farrar P Cascales ÁF Sauro S. Bonding performance and interfacial adaptation of modern bulk-fill restorative composites after aging in artificial saliva: an *in vitro* study. Clin Oral Investig. (2024) 28(2):132. 10.1007/s00784-024-05525-5 38308668

[5] Martignon S Cortes A Douglas GVA Newton JT Pitts NB Avila V Cariescare international adapted for the pandemic in children: caries OUT multicentre single-group interventional study protocol. BMC oral Health. (2021) 21(1):1–13. 10.1186/s12903-021-01674-1 34210281 PMC8248759

[6] Doméjean S White JM Featherstone JDB. Validation of the CDA CAMBRA caries risk assessment–a six-year retrospective study. J Calif Dent Assoc. (2011) 39(10):709–15. 10.1080/19424396.2011.12221948 22132582

[7] Martignon S Pitts NB Goffin G Mazevet M Douglas GVA Newton JT Cariescare practice guide: consensus on evidence into practice. Br Dent J. (2019) 227(5):353–62. 10.1038/s41415-019-0678-8 31520031

[8] Slimani A Terrer E Manton DJ Tassery H. Carious lesion detection technologies: factual clinical approaches. Br Dent J. (2020) 229(7):432–42. 10.1038/s41415-020-2116-3 33037363

[9] Ismail AI Sohn W Tellez M Amaya A Sen A Hasson H The international caries detection and assessment system (ICDAS): an integrated system for measuring dental caries. Community Dent Oral Epidemiol. (2007) 35(3):170–8. 10.1111/j.1600-0528.2007.00347.x 17518963

[10] Pitts N Ekstrand K, ICDAS Foundation. International caries detection and assessment system (ICDAS) and its international caries classification and management system (ICCMS) - methods for staging of the caries process and enabling dentists to manage caries. Community Dent Oral Epidemiol. (2013) 41(1):e41–52. 10.1111/cdoe.12025 24916677

[11] Tassery H Levallois B Terrer E Manton D Otsuki M Koubi S Use of new minimum intervention dentistry technologies in caries management. Aust Dent J. (2013) 58(s1):40–59. 10.1111/adj.12049 23721337

[12] Koubi S Tassery H. Minimally invasive dentistry using sonic and ultra-sonic devices in ultraconservative class 2 restorations. J Contemp Dent Pract. (2008) 9(2):155–65. 10.5005/jcdp-9-2-155 18264538

[13] Al Marzooq F Al Kawas S Al Bayat S Sayyar F Ishaq H Nasralla H Infection control in the dental clinics: probiotic-based cleaning as an alternative to chemical disinfection. J Infect Public Health. (2019) 12(1):128. 10.1016/j.jiph.2018.10.074

[14] Chun L Yan-hong L Juan L. Application of probiotics for dental caries prevention in children. 口腔疾病防治. (2016) 24:558–60. 10.12016/j.issn.2096-1456.2016.09.013

[15] Schwendicke F Splieth CH Bottenberg P Breschi L Campus G Domejean S How to intervene in the caries process in adults: proximal and secondary caries? An EFCD-ORCA-DGZ expert delphi consensus statement. Clin Oral Investig. (2020) 24(9):3315–21. 10.1007/s00784-020-03431-0 32643090

[16] Featherstone JDB Domejean-Orliaguet S Jenson L Wolff M Young DA. Caries risk assessment in practice for age 6 through adult. J Calif Dent Assoc. (2007) 35(10):703–13. 10.1080/19424396.2007.12221276 18044378

[17] Sauro S Makeeva I Faus-Matoses V Foschi F Giovarruscio M Maciel Pires P Effects of ions-releasing restorative materials on the dentine bonding longevity of modern universal adhesives after load-cycle and prolonged artificial Saliva aging. Materials. (2019) 12(5):722. 10.3390/ma12050722 30832247 PMC6427106

[18] Slimani A Sauro S Gatón Hernández P Gurgan S Turkun LS Miletic I Commercially available Ion-releasing dental materials and cavitated carious lesions: clinical treatment options. Materials (Basel). (2021) 14(21):6272. 10.3390/ma14216272 34771800 PMC8585007

[19] Alleman DS Magne P. A systematic approach to deep caries removal end points: the peripheral seal concept in adhesive dentistry. Quintessence Int. (2012) 43:197–208. 22299120

[20] Schwendicke F Frencken JE Bjørndal L Maltz M Manton DJ Ricketts D Managing carious lesions: consensus recommendations on carious tissue removal. Adv Dent Res. (2016) 28(2):58–67. 10.1177/0022034516639271 27099358

[21] Tassery H Miletic I Turkun LS Sauro S Gurgan S Banerjee A Preventive management of carious lesions: from non-invasive to micro-invasive operative interventions. Br Dent J. (2024) 236(8):603–10. 10.1038/s41415-024-7292-0 38671111

[22] Sauro S Carvalho RM Ferracane J. The rise of advanced bioactive restorative materials: are they redefining operative dentistry? Dent Mater. (2025) 41(11):1411–29. 10.1016/j.dental.2025.08.003 40803933

[23] Albelasy EH Hamama HH Chew HP Montaser M Mahmoud SH. Secondary caries and marginal adaptation of ion-releasing versus resin composite restorations: a systematic review and meta-analysis of randomized clinical trials. Sci Rep. (2022) 12(1):19244–17. 10.1038/s41598-022-19622-6 36357453 PMC9649593

[24] Adsul PS Dhawan P Tuli A Khanduri N Singh A. Evaluation and comparison of physical properties of cention N with other restorative materials in artificial Saliva: an *in vitro* study. Int J Clin Pediatr Dent. (2022) 15(3):350–5. 10.5005/jp-journals-10005-2383 35991795 PMC9357546

[25] Justen M Scheck D Münchow EA Jardim JJ. Is cention-N comparable to other direct dental restorative materials? A systematic review with network meta-analysis of *in vitro* studies. Dent Mater. (2024) 40(9):1341–52. 10.1016/j.dental.2024.06.014 38880724

[26] Pai D Anirudhmaadhava PA Ginjupalli K. *In Vitro* evaluation of mechanical properties of cention N and its comparison with resin modified glass ionomer cement (RMGIC) restorative material as used in primary teeth. The Scientific World Journal. (2024) 2024:1–7. 10.1155/2024/9420336 PMC10776193 38205145

[27] Du H Wang Z Long S Li Y Yang D. The advancement of nanosystems for drug delivery in the prevention and treatment of dental caries. Front Cell Infect Microbiol. (2025) 15:1546816. 10.3389/fcimb.2025.1546816 40007606 PMC11850577

[28] Arbildo-Vega HI Cruzado-Oliva FH Coronel-Zubiate FT Luján-Valencia SA Meza-Málaga JM Aguirre-Ipenza R Clinical effectiveness of Ion-releasing restorations versus composite restorations in dental restorations: systematic review and meta-analysis. Dentistry Journal. (2024) 12(6):158. 10.3390/dj12060158 38920859 PMC11203382

[29] Slimani A Giraudeau N Levallois B Cuisinier F Tassery H Terrer E Performance of fluorescence-based systems in early caries detection: a public health issue. J Contemp Dent Pract. (2019) 20(10):1126–32. 10.5005/jp-journals-10024-2665 31883244

[30] Zeitouny M Cuisinier F Tassery H Fayyad-Kazan H. The efficacy of soprolife ® in detecting *in vitro* remineralization of early caries lesions. J Oral Maxillofac Res. (2020) 11(2):e6. 10.5037/jomr.2020.11206 32760479 PMC7393931

[31] Moecke SE Silva AGDCS Andrade ACM Borges AB Torres CRG. Efficacy of S-PRG filler varnishes on enamel caries remineralization. J Dent. (2022) 119:104074. 10.1016/j.jdent.2022.104074 35218877

[32] Abdullah N Al Marzooq F Mohamad S Abd Rahman N Rani KGA Chi Ngo H The antibacterial efficacy of silver diamine fluoride (SDF) is not modulated by potassium iodide (KI) supplements: a study on *in-situ* plaque biofilms using viability real-time PCR with propidium monoazide. PLoS One. (2020) 15(11):e0241519. 10.1371/journal.pone.0241519 33141868 PMC7608867

[33] Abdulrahim R Splieth CH Mourad MS Vielhauer A Khole MR Santamaría RM. Silver diamine fluoride renaissance in paediatric dentistry: a 24-month retrospective and cross-sectional analysis. Medicina (B Aires). (2024) 60(1):16. 10.3390/medicina60010016 PMC10820628 38276050

[34] de Almeida LDFD Cavalcanti YW Valença AMG. *In vitro* antibacterial activity of silver diamine fluoride in different concentrations. Acta Odontológica Latinoamericana. (2011) 24:127–31. 22165309

[35] Doméjean S Ducamp R Léger S Holmgren C. Resin infiltration of non-cavitated caries lesions: a systematic review. Med Princ Pract. (2015) 24(3):216–21. 10.1159/000371709 25661012 PMC5588225

[36] Alkilzy M Tarabaih A Santamaria RM Splieth CH. Self-assembling peptide P11-4 and fluoride for regenerating enamel. J Dent Res. (2018) 97(2):148–54. 10.1177/0022034517730531 28892645 PMC6429572

[37] Alkilzy M Santamaria RM Schmoeckel J Splieth CH. Treatment of carious lesions using self-assembling peptides. Adv Dent Res. (2018) 29(1):42–7. 10.1177/0022034517737025 29355413

[38] Peng JJ-Y Botelho MG Matinlinna JP. Silver compounds used in dentistry for caries management: a review. J Dent. (2012) 40(7):531–41. 10.1016/j.jdent.2012.03.009 22484380

[39] Beltrán-Aguilar ED. Silver diamine fluoride (SDF) may be better than fluoride varnish and no treatment in arresting and preventing cavitated carious lesions. J Evid Based Dent Pract. (2010) 10(2):122–4. 10.1016/j.jebdp.2010.02.014 20466328

[40] Lall M. Is SDF better than the SMART (silver modified atraumatic restorative technique) in the management of molar incisor hypomineralisation molars with initial caries? Evid Based Dent. (2024) 25:162–3. 10.1038/s41432-024-01062-y 39256484

[41] Knight G. Evidence-based research links SDF treatments to numerous oral health benefits. Br Dent J. (2022) 233(12):1053. 10.1038/s41415-022-5373-5

[42] Keeper JH Kibbe LJ Thakkar-Samtani M Heaton LJ Desrosiers C Vela K Systematic review and meta-analysis on the effect of self-assembling peptide P11-4 on arrest, cavitation, and progression of initial caries lesions. J Am Dent Assoc. (2023) 154(7):580–591.e11. 10.1016/j.adaj.2023.03.014 37245138

[43] Jablonski-Momeni A Heinzel-Gutenbrunner M. Efficacy of the self-assembling peptide P11-4 in constructing a remineralization scaffold on artificially-induced enamel lesions on smooth surfaces. J Orofac Orthop. (2014) 75(3):175–90. 10.1007/s00056-014-0211-2 24825830

[44] Bourouni S Dritsas K Kloukos D Wierichs RJ. Efficacy of resin infiltration to mask post-orthodontic or non-post-orthodontic white spot lesions or fluorosis — a systematic review and meta-analysis. Clin Oral Invest. (2021) 25(8):4711–9. 10.1007/s00784-021-03931-7 PMC8342329 34106348

[45] Alkilzy M Qadri G Splieth CH Santamaría RM. Biomimetic enamel regeneration using self-assembling peptide P11-4. Biomimetics. (2023) 8(3):290. 10.3390/biomimetics8030290 37504178 PMC10807035

[46] Dawasaz AA Togoo RA Mahmood Z Azlina A Thirumulu Ponnuraj K. Effectiveness of self-assembling peptide (P11-4) in dental hard tissue conditions: a comprehensive review. Polymers. (2022) 14(4):792. 10.3390/polym14040792 35215706 PMC8879648

[47] Alsabek L Al-Nerabieah Z Bshara N Comisi JC. Retention and remineralization effect of moisture tolerant resin-based sealant and glass ionomer sealant on non-cavitated pit and fissure caries: randomized controlled clinical trial. J Dent. (2019) 86:69–74. 10.1016/j.jdent.2019.05.027 31136817

[48] Muller-Bolla M Lupi-Pégurier L Bardakjian H Velly AM. Effectiveness of school-based dental sealant programs among children from low-income backgrounds in France: a pragmatic randomized clinical trial. Community Dent Oral Epidemiol. (2013) 41(3):232–41. 10.1111/cdoe.12011 23072366

[49] Muller-Bolla M Courson F Lupi-Pégurier L Tardieu C Mohit S Staccini P Effectiveness of resin-based sealants with and without fluoride placed in a high caries risk population: multicentric 2-year randomized clinical trial. Caries Res. (2018) 52(4):312–22. 10.1159/000486426 29495020

[50] Ahmed B Wafaie RA Hamama HH Mahmoud SH. 3-year Randomized clinical trial to evaluate the performance of posterior composite restorations lined with ion-releasing materials. Sci Rep. (2024) 14(1):4942. 10.1038/s41598-024-55329-6 38418863 PMC10902344

[51] Deery C. Strong evidence for the effectiveness of resin based sealants. Evid Based Dent. (2013) 14(3):69–70. 10.1038/sj.ebd.6400945 24071670

[52] Wright JT Crall JJ Fontana M Gillette EJ Nový BB Dhar V Evidence-based clinical practice guideline for the use of pit-and-fissure sealants: a report of the American dental association and the American academy of pediatric dentistry. The Journal of the American Dental Association. (2016) 147:672–682.e12. 10.1016/j.adaj.2016.06.001 27470525

[53] Alsabek L Al-Hakeem A Alagha MA Comisi JC. Efficacy of hydrophilic resin-based sealant: a systematic review and meta-analysis. J Dent. (2021) 114:103816. 10.1016/j.jdent.2021.103816 34560227

[54] François P Greenwall-Cohen J Goff SL Ruscassier N Attal J-P Dursun E. Shear bond strength and interfacial analysis of high-viscosity glass ionomer cement bonded to dentin with protocols including silver diamine fluoride. J Oral Sci. (2020) 62(4):444–8. 10.2334/josnusd.20-0065 32879159

[55] Kunert M Piwonski I Hardan L Bourgi R Sauro S Inchingolo F Dentine remineralisation induced by “bioactive” materials through mineral deposition: an *in vitro* study. Nanomaterials. (2024) 14(3):274. 10.3390/nano14030274 38334546 PMC10857417

[56] Maciel Pires P Dávila-Sánchez A Faus-Matoses V Nuñez Martí JM Lo Muzio L Sauro S. Bonding performance and ultramorphology of the resin-dentine interface of contemporary universal adhesives. Clin Oral Investig. (2022) 26(6):4391–405. 10.1007/s00784-022-04402-3 35149904

[57] Türkün M Türkün LS Kalender A. Effect of cavity disinfectants on the sealing ability of nonrinsing dentin-bonding resins. Quintessence Int. (2004) 35:469–76. 15202592

[58] Sharma S Logani A Shah N. Comparative efficacy of photo-activated disinfection and calcium hydroxide for disinfection of remaining carious dentin in deep cavities: a clinical study. Restor Dent Endod. (2014) 39(3):195–200. 10.5395/rde.2014.39.3.195 25110643 PMC4125583

[59] Araújo NC Fontana CR Bagnato VS Gerbi MEM. Photodynamic antimicrobial therapy of curcumin in biofilms and carious dentine. Lasers Med Sci. (2014) 29(2):629–35. 10.1007/s10103-013-1369-3 23793414

[60] Ismail MMM Al Haidar AHMJ. Impact of brix 3000 and conventional restorative treatment on pain reaction during caries removal among group of children in Baghdad city. J Coll Dent. (2019) 31(2):7–13. 10.26477/jbcd.v31i2.2617

[61] Chittem J Sajjan G Varma K. Comparative evaluation of microshear bond strength of the caries-affected dentinal surface treated with conventional method and chemomechanical method (papain). J Conserv Dent. (2015) 18(5):369–73. 10.4103/0972-0707.164034 26430299 PMC4578180

[62] Hamama H Yiu C Burrow M. Effect of silver diamine fluoride and potassium iodide on residual bacteria in dentinal tubules. Aust Dent J. (2015) 60(1):80–7. 10.1111/adj.12276 25721282

[63] Hameed H Babu BP Sagir VMM Chiriyath KJ Mathias J Shaji AP. Microleakage in resin composite restoration following antimicrobial Pre-treatments with 2% chlorhexidine and clearfil protect bond. J Int Oral Health. (2015) 7:71–6. PMID: 26229374 PMCID: PMC4513780 26229374 PMC4513780

[64] Imazato S Kuramoto A Takahashi Y Ebisu S Peters MC. *In vitro* antibacterial effects of the dentin primer of clearfil protect bond. Dent Mater. (2006) 22(6):527–32. 10.1016/j.dental.2005.05.009 16198404

[65] Imazato S Walls AWG Kuramoto A Ebisu S. Penetration of an antibacterial dentine-bonding system into demineralized human root dentine *in vitro*. Eur J Oral Sci. (2002) 110(2):168–74. 10.1034/j.1600-0722.2002.11221.x 12013562

[66] Pourhajibagher M Bahador A. Physico-mechanical properties, antimicrobial activities, and anti-biofilm potencies of orthodontic adhesive containing cerium oxide nanoparticles against Streptococcus mutans. Folia Med. (2022) 64(2):252–9. 10.3897/folmed.64.e60418 35851777

[67] Gutiérrez MF Aliaga-Gálvez R Ñaupari-Villasante R Fernández E Loguercio AD. Universal dental adhesives containing zinc oxide and copper nanoparticles improve interface on caries-affected dentin after 2 years: *in vitro* study. J Esthet Restor Dent. (2025):jerd.70095. 10.1111/jerd.70095 PMC13069364 41468243

[68] Hanzen TA Gutiérrez MF de Paris Matos T Mara de Paula A Figueredo de Siqueira FS Millán Cardenas AF A universal dental adhesive containing copper nanoparticles stabilizes the hybrid layer in eroded dentin after 1 year. Int J Adhes Adhes. (2022) 113:103041. 10.1016/j.ijadhadh.2021.103041

[69] Vidal O de Paris Matos T Núñez A Méndez-Bauer L Sutil E Ñaupari-Villasante R A universal adhesive containing copper nanoparticles improves the stability of hybrid layer in a cariogenic oral environment: an *in situ* study. J Mech Behav Biomed Mater. (2022) 126:105017. 10.1016/j.jmbbm.2021.105017 34894497

[70] Tay FR Pashley DH. Biomimetic remineralization of resin-bonded acid-etched dentin. J Dent Res. (2009) 88(8):719–24. 10.1177/0022034509341826 19734458 PMC2874868

[71] Schwendicke F Al-Abdi A Pascual Moscardó A Ferrando Cascales A Sauro S. Remineralization effects of conventional and experimental ion-releasing materials in chemically or bacterially-induced dentin caries lesions. Dent Mater. (2019) 35(5):772–9. 10.1016/j.dental.2019.02.021 30853209

[72] Türkün L Kanik Ö. A prospective six-year clinical study evaluating reinforced glass ionomer cements with resin coating on posterior teeth: quo vadis? Oper Dent. (2016) 41(6):587–98. 10.2341/15-331-C 27571238

[73] Mickenautsch S. Are high-viscosity glass-ionomer cements inferior to silver amalgam as restorative materials for permanent posterior teeth? A Bayesian analysis. BMC Oral Health. (2015) 15(1):118. 10.1186/s12903-015-0108-5 26449638 PMC4599034

[74] Mickenautsch S Yengopal V. Failure rate of direct high-viscosity glass-ionomer versus hybrid resin composite restorations in posterior permanent teeth - a systematic review. TODENTJ. (2015) 9(1):438–48. 10.2174/1874210601509010438 PMC4768657 26962372

[75] Oz FD Meral E Gurgan S. Clinical performance of an alkasite-based bioactive restorative in class II cavities: a randomized clinical trial. J Appl Oral Sci. (2023) 31:e20230025. 10.1590/1678-7757-2023-0025 37377309 PMC10343945

[76] Htet K Hiraishi N Sanon K Ubolsaard P Sone KP Shimada Y. Effect of zinc-releasing glass ionomer cement on preventing dentin demineralization. J Dent. (2025) 156:105718. 10.1016/j.jdent.2025.105718 40139424

[77] Manente R da Silva Mira PC Milori Corona SA. Bioactive materials with S-PRG filler in paediatric dentistry: a scoping review. Eur Arch Paediatr Dent. (2025). 10.1007/s40368-025-01077-8 40637825

[78] Ferreira NF de Oliveira Alves R de Toledo PTA Peres GR Danelon M Guiotti AM Bioactive S-PRG materials in dental applications: a clinical evidence–based scoping review. Dent Mater. (2026) 42(3):512–32. 10.1016/j.dental.2025.11.012 41309331

[79] Sunami A Inokoshi M Tamura M Takahashi R Kanazawa M. Shear bond strength of relining material containing nano S-PRG filler. Dent Mater. (2025) 41:e33–4. 10.1016/j.dental.2025.03.086

[80] Preusse PJ Winter J Amend S Roggendorf MJ Dudek M-C Krämer N Class II resin composite restorations—tunnel vs. Box-only *in vitro* and *in vivo*. Clin Oral Invest. (2021) 25(2):737–44. 10.1007/s00784-020-03649-y PMC8364904 33169273

[81] Knight GM. The tunnel restoration – nine years of clinical experience using capsulated glass ionomer cements. Case report. Aust Dent J. (1992) 37(4):245–51. 10.1111/j.1834-7819.1992.tb04738.x 1444941

[82] Fruits TJ Knapp JA Khajotia SS. Microleakage in the proximal walls of direct and indirect posterior resin slot restorations. Oper Dent. (2006) 31(6):719–27. 10.2341/05-148 17153983

[83] Moosavi H Abedini S. The effect of various placement techniques on the microhardness of class II (slot) resin composite restorations. J Contemp Dent Pract. (2009) 10(6):9–16. 10.5005/jcdp-10-6-9 19838605

[84] Rathnam A Nidhi M Shigli A Indushekar K. Comparative evaluation of slot versus dovetail design in class III composite restorations in primary anterior teeth. Contemp Clin Dent. (2010) 1(1):6. 10.4103/0976-237X.62511 22114369 PMC3220074

[85] Gupta A. SDI’s stela self-cure bulk fill flowable self cure restorative delivers stress-free bulk-fill restorations. Dental Products Report. (2024) 58:27.

[86] Loguercio AD Carpio-Salvatierra B Ñaupari-Villasante R Wendlinger M Armas-Vega A Cavagnaro S Clinical evaluation of a new chemically-cured bulk-fill composite in posterior restorations: 6-month multicenter double-blind randomized clinical trial. J Dent. (2024) 149:105246. 10.1016/j.jdent.2024.105246 39025426

[87] Francois P Fouquet V Attal JP Dursun E. Commercially available fluoride-releasing restorative materials: a review and a proposal for classification. Materials. (2020) 13(10):2313. 10.3390/ma13102313 32443424 PMC7287768

[88] Duncan HF Galler KM Tomson PL Simon S El-Karim I Kundzina R European society of endodontology position statement: management of deep caries and the exposed pulp. Int Endod J. (2019) 52(7):923–34. 10.1111/iej.13080 30664240

[89] Albelasy E Raghip A Ismail H. Internal adaptation and micromorphological analysis of a new self-cure resin composite. J Clin Exp Dent. (2025) 17:e912–9. 10.4317/jced.62900 40950526 PMC12424602

[90] Mantri CA. An in vitro assessment of the antibacterial, biocompatibility, and mineralization inducing potential of biodentine® Xp (Proquest dissertations & theses). (2023). Available online at: https://go.exlibris.link/cZt8qwgf

[91] Duncan HF Kirkevang L Peters OA El-Karim I Krastl G Del Fabbro M Treatment of pulpal and apical disease: the European society of endodontology (ESE) S3-level clinical practice guideline. Int Endod J. (2023) 56(S3):238–95. 10.1111/iej.13974 37772327

[92] Duncan HF El-Karim I. Endodontic S3-level clinical practice guidelines: the European society of endodontology process and recommendations: endodontic S3-level clinical practice guidelines: the European society of endodontology process and recommendations. Br Dent J. (2025) 238(7):580–6. 10.1038/s41415-025-8335-x 40217051 PMC11991915

[93] Niu JY Ge KX Yin IX Zhang OL Zhao IS Chu CH. Next-gen restorative materials to revolutionise smiles. Bioengineering. (2026) 13(2):143. 10.3390/bioengineering13020143 41749683 PMC12937712

[94] Alamoudi NA Aldhali AA Alolyet AM ALjohani EH Huraib AG Alotaibi FF Emerging trends in restorative dental materials: review article. World J Environ Biosci. (2025) 14(4):44–53. 10.51847/Zo7BPz0deN

[95] Abozaid D Azab A Bahnsawy MA Eldebawy M Ayad A Soomro R Bioactive restorative materials in dentistry: a comprehensive review of mechanisms, clinical applications, and future directions. Odontology. (2025). 10.1007/s10266-025-01162-w PMC13053330 40819015

