# Supplementary material for: Bioactive and Ion-releasing materials in minimum intervention dentistry: a clinical pathway from prevention to restorative treatment
Source: Front Dent Med. 2026 Apr 8;7:1739208. doi: 10.3389/fdmed.2026.1739208 (PMC13099902; doi:10.3389/fdmed.2026.1739208)
Supplement: Supplementary file 1 [file Datasheet1.pdf]

Supplementary materials.

Table 9 Professional remineralization or at home and preventive products used for non-invasive and micro-invasive treatments.

Materials	Occlusal pits and fissures lesions	Proximal lesions	Facial or lingual smooth surface lesions	Root surface lesions	Commercially available products
**NI at-home**<br>Fluoride gels /+ CPP-ACP / toothpastes with high concentration of fluoride (5000ppm).	1.23% APF gel (Application every 3-6 months), daily toothbrushing with specific toothpastes. CPP-ACP: has proven to be an adjunctive treatment to fluoride therapy in the management of early carious lesions. CPP-ACP-based remineralization technology allows high concentrations of calcium and phosphate ions and can bind to the biofilm with buffering effect.			Daily toothbrushing with specific toothpastes 5,000 parts per million fluoride (1.1% NaF) if caries susceptibility.	Gel: APF (Medicom, Canada), Toothpastes: Duraphat®5000 (Colgate, USA). GC Tooth mousse® + 900ppm fluorid, Recaldent® (GC, Japan).
**NI at home**<br>0.2% NaF: Mouth rinses often associated with xylitol.	0.2% NaF mouth rinse (10ml once a week).				PreviDent rinse ® (Colgate, USA), X-PUR OPTI-Rinse ®0.2% (Canada), Flux Dry Mouth Rinse™ (Karo Pharma AB, Sweden)

**NI at home.**<br><br>Nano-hydroxyapatite (nHA).<br><br>Remin Pro®, X-Pure® Remin®, CariFree® CTx3 Gel	Products: Remin Pro® is a water-based cream containing hydroxyapatite, fluorides sodium (1450ppm) and xylitol (3mn application after tooth brushing, not to be used under 12 years old). X-Pure® Remin® X is a toothpaste containing 10% nano-hydroxyapatite and 10%Xylitol. It contains no Fluorine or Sodium Lauryl Sulfate and has a neutral pH. Main Indications: Patient at moderate caries risk and hypersensitivity. Protocol: Use instead of conventional toothpaste 2 to 3 times a day, apply on a soft toothbrush paste the size of a pea and brush for 2min. CariFree® CTx3 Gel is a refreshing, non-abrasive, fluoride-free dental gel that combines bioavailable hydroxyapatite nanocrystals, xylitol and the unique pH neutralisation technology of CariFree®. Use instead of conventional toothpaste 2 to 3 times a day. For all products, the patient should spit without rinsing, wait at least 30 minutes before eating or drinking.
Tricalcium phosphate. NI at home-Clinpro® 5000, NI at hone-Clinpro® Tooth Cream, NI professional - Clinpro®White,Varnish	Clinpro® 5000 is a prescription anti-cavity toothpaste, use instead of conventional toothpaste containing fluoride sodium (5000ppm) and functionalized Tricalcium Phosphate (fTCP). It contains fluoride, calcium and phosphate. This product is not suitable for children under 6 years of age. Clinpro® Tooth Cream is a water-based cream, use instead of conventional toothpaste containing sodium fluoride (950ppm) and functionalized Tricalcium Phosphate (fTCP). It contains fluoride, calcium and phosphate, Clinpro® White Varnish is a colourless varnish containing 22,600 ppm fluoride sodium, functionalized TCP and xylitol. 1application every 6 month. Efficacy of fTCP seems probably related to the action of brushing.
**NI at home**<br><br>Calcium sodium phosphosilicate or Novamin®<br><br>BioMin® F, BioMin® C. Sérum Expert	BioMin® F is an innovative toothpaste contains a low level of fluoride (530ppm) with bioactive glass which dissolve gradually (12h) in the saliva, increasing the pH and releasing minerals that guarantee a healthy tooth surface. BioMin® C is the version without fluoride. Sérum Expert Regenerate Enamel Scienc® is a patented NR-5® technology which significantly reduces the dental hypersensitivity, increases the hardness of the enamel when combined with the Expert toothpaste, for a

Regenerate Enamel Science®.	remineralization of 82% of the enamel after 3 days. The serum contains calcium and sodium phosphosilicates and the activating gel contains sodium fluoride (600 ppm). 3mn application on bite tray (3 days per month).				
**NI professional application.** Fluoride Varnish, + CPP-ACP, 5% + CXP® ***Calcium Xylitol et Phosphate)****	All kind of 5% NaF varnish (application every 3-6 months, 22600 ppm fluoride).				Fluoride varnish: Embrace® varnish* (Pulpdent, USA) , Profluorid® + Biomin (Voco, Germany) Fluor Protector S™. (Ivoclar, Schain), Duraphat® (Colgate, USA). Fluoride varnish with CPP-ACP: MI varnish® (GC, Japan), Enamel Pro® with ACP (Premier , USA)
**Micro-invasive professional application.**	Poor data for the occlusal area	If micro-invasive therapies:	If micro-invasive therapies:	Poor data	ICON® (DMG, Germany)

Resin infiltration. Dental dam mandatory. Micro invasive therapy.		Resin infiltration	Resin infiltration.		
**NI or micro-invasive (if etching) professional application.**<br>38% Silver diammine fluoride. Micro invasive therapy if combined with etching procedure.	Annual application.	Annual application after having strongly separated the teeth.	Annual application.	If active root caries 38% SDF solution (annual application)<br>. 1% chlorhexidine plus 1% thymol varnish (very low recommendations for CHX) (Application every 3-6 months).	Riva Star® (SDI, Australia), Cariestop® (Biodinâmica, Brazil), FAgamin® (Tedequim, Argentina), Advantage Arrest® (Elevate Oral Care, USA),e-SDF® (Kids-E-Dental, India), Saforide® (Toyo Seiyaku Kasei Co. Ltd, Japan).
**Micro-invasive professional application.**<br>Self-peptide P11-4. Curodont®Curodont Protect®Curolox® Technology.	Indications: occlusal caries, active white spots in patients with increased risk, secondary caries around already existing restorations, hypersensitivity of the teeth. Clinical protocol: airflow cleaning of the area, remove organic debris from decay by cleaning it for 20 seconds with sodium hypochlorite 2%, for the interproximal areas dental floss can be used impregnated or a sponge stick. Non-organic debris is removed with phosphoric acid, for 20s too. Remove sodium hypochlorite and acid with water and dry the tooth.				

	The freeze-dried Curodont Repair® is supplied in small vials, is dissolved in 0.05 ml of water, sterile preference, a drop is applied to the lesion, wait 5min until the drop is gone. It is advisable to bring a source of topical fluoride such as Curodont®Protect. Curodont® Protect is a gel manufactured for occupational prophylaxis containing Curolox® Technology and monofluorophosphate at 900ppm. It can be applied at home as the Curodont ® D'SENZ in case of dental hypersensitivity. This product can be applied several times with intervals of 3 to 6 months until complete remineralization. The new hydroxyapatite crystals generated by SAP11-4 do not have a prismatic structure, but exhibit a "fan-like" structure, as they are arranged tangentially to the matrix fibers and in comparison with TCPF varnish, SAP11-4 peptide demonstrated greater remineralizing.
**Levels of certainty and strength for all.**	Pre and pro-biotics were not described as the levels of certainty for these products were too low. Others products described ranked between low or moderate level of certainty.

Table 10 Optional restorative materials for micro, mini and invasive clinical applications

Types of materials	Bioactive properties	Biological effects	Drawbacks	Commercially available products
**Conventional GICs.**	Ions release: F, Ca and Al released. Formation of polyalkenoate salts with a interdiffusion zone and calcium polycarbonate. *Flow GIC with high fluorid released.	Antibacterial effects, hard tissues remineralization, bulk-fill reaction.	Long setting reaction, low wear resistance, and low aesthetic.	IonoStar Plus®, IonoFil®, Aqua Ionofil Plus® (VOCO, Germany) , Ketac Universal®, Ketac Fil Plus® (3M ESPE, St Paul, MN, USA). Riva Self Cure® (SDI, Australia). Riva protect®*(SDI, Australia). GC Fuji II® (GC, Tokyo, Japan).
**High viscosity GICs.**	Ions release and reload: F, Ca, Al. Formation of polyalkenoate salts with a interdiffusion zone and calcium polycarbonate.	Antibacterial effects, hard tissues remineralization, bulk-fill reaction.	Short setting reaction, high viscosity depend of products.	Fuji IX Fast®, Fuji IX GP® , Fuji IX Extra® (GC, Tokyo, Japan). Chemfil Rock (Dentsply, Germany). IonoStar Molar®, Ionofil Molar®, Ionofil Molar AC® Quick (VOCO, Germany), Ketac Molar®, Ketac Molar Quick®, (3M ESPE, St Paul, MN, USA). Riva self-cure HV® (SDI, Australia).

**RMGICs and HV-RMGICs.**	Fluorid, calcium and aluminium released, formation of polyalkenoate salts with interdiffusion zone, Formation of calcium polycarbonate.	Facilitates tissues remineralisation, antibacterial effects.	Not a true bulk-fil reaction, no covalent or ionic bond between the 2 networks, absorption of water due to residual HEMA, low wear resistance, except HV-RMGICs.	RMGICs: Ionolux® (VOCO, Germany) Photac Fil Quick Aplicap®, Ketac Nano® , Vitremer® (3M ESPE, St Paul, MN, USA), Riva Light Cure UV®, Riva Light cure and Riva light-cure HV® (SDI, Australia), Fuji II LC GC®(Tokyo, Japan).
**Mineral-enriched composites.**	Releasing of Fluorure, powder containing fluoro-alumino silicate particles and polyacid components.	Material can reduce the degradation during load cycling and/or prolonged storage in artificial saliva of the hybrid layer created with modern universal adhesive applied in etch and rinse mode.	Lack of studies for Re-gen products	A) Activa®, Activa liner®, Presto®, Activa Bulkfil dual® (Pulpdent, USA).<br>B) Re-Gen Flowable® Composite, Re-Gen Bulk Fill Composite (Apex, USA).<br>Replica bulkfil ® (Parkwell, USA).

**Mineral-enriched self-adhesive composite.**	High molecular weight polyacrylic acid functionalized with polymerizable groups (MOPOS). Photo and chemo activation. Fluorure and aluminium ions released . No adhesive system combined.	Released of Fluoride, Calcium and Aluminium.	Very high viscosity, lack of evidence as new product, short time setting. Lack of studies.	Surfil 1® Self adhesive hybride composite: New GIC family. (Dentsply, USA).
**Mineral-enriched Alkasite.**	No acid/base reaction. Alkaline glass filler reactiong with water. In this SiO2 are 3 salts connected together (Na2O, CaO, CaF2). In contact with the saliva these salts are dissolved and released Ca, F and OH ions depend of the	Hydroxy ion: Regulates the pH-value during acid attack and prevent demineralization. Buffering ability at pH 5.7. Fluoride & calcium: To prevent demineralization of the tooth substrate. Forming apaptite in vitro on dentine at pH 7 if	Very high viscosity, lack of evidence as new product, short time setting. Lack of studies.	Cention® Forte (Dual) (Ivoclar-Vivadent, Liechstenstein).

	pH. Combine with a specific Primer.	phosphate available.		
**Self-cure material (automix or Capsule) with Primer.**	Self-cured material, starting from the bottom of the cavity, thanks to the primer.	Amalgam's alternative.	No long term studies.	Stela® (SDI, Australia).
**Calcium silicate-based cements.**		Vital pulp therapy.	Time setting. Liner or temporary restauration.	Biodentine® (Septodont, France).
**Resin-modified MTA: TheraCal LC® (Bisco, USA).**		Vital pulp therapy. Easy to use, dentine bridge formation.		MTA Plus®, Neo MTA (Avalon Biomed Inc., USA), Endosequence BC sealer® (Brassler, USA) Generex A (Dentsply, USA), ProRoot MTA® (Dentsply, USA), Angelus MTA Bio® (Angelus, Brazil). BioAggregate® (Innovative BioCeramix), RetroMTA BioMTA, (Republic of Korea)

**Silver Diammine Fluoride.**	Silver is an anti-microbial agent. Fluoride has bacteriostatic effect and potassium iodide used in conjunction with SDF provides a powerful antimicrobial effect as well as reducing potential staining of teeth.	High caries risk, geriatric dentistry. Apply before HVGIC. Can be combined with enzyme or chemomechanical conditioning.	Discoloration	Riva Star (SDI, Australia), Cariestop (Biodinâmica, Brazil), FAgamin (Tedequim, Argentina), , Advantage Arrest (Elevate Oral Care, USA),e-SDF (Kids-E-Dental, India), Saforide (Toyo Seiyaku Kasei Co. Ltd, Japan).
**Resin-modified glass-ionomer.**	Ionglass™ fillers, which contain fluoro-aluminosilicate glass for radiopacity, and fluoride released.	With Composite restoration alone or combined with RMGIC as dentine substitute.		Riva Bond LC™ (SDI, Australia), FUJI Bond LC™ (GC, Japan).
**Adhesive with chlorhexidine (CHX).**	Release of CHX.	Antibacterial effects, stabilization of the hybrid layer, anti MMPs effects.	Time limiting effects.	Peak adhesive (Ultradent, USA).

Table 11 OCEBM ranking.

Levels	Descriptions
**L1a,b**	Systematic review of randomised controlled trials (RCTs) or high-quality RCTs with narrow confidence intervals. a: Systematic review (with homogeneity) of Level 1b studies or better. b: individual randomised controlled trial (with a narrow confidence interval).
**L2**	Randomised controlled trial (RCT) or observational study with dramatic effect.
**L3**	Non-randomised controlled cohort/follow-up study.
**L4**	Case-series, case-control, or historically controlled studies.
**L5**	Mechanism-based reasoning or expert opinion.
